# Alterations in Polyadenylation and Its Implications for Endocrine Disease

**DOI:** 10.3389/fendo.2013.00053

**Published:** 2013-05-08

**Authors:** Anders Rehfeld, Mireya Plass, Anders Krogh, Lennart Friis-Hansen

**Affiliations:** ^1^Genomic Medicine, Rigshospitalet, Copenhagen University HospitalCopenhagen, Denmark; ^2^Department of Biology, The Bioinformatics Centre, University of CopenhagenCopenhagen, Denmark

**Keywords:** polyadenylation, gene expression regulation, diabetes, IGF-1, pre-eclampsia, RET, antisense elements

## Abstract

**Introduction:** Polyadenylation is the process in which the pre-mRNA is cleaved at the poly(A) site and a poly(A) tail is added – a process necessary for normal mRNA formation. Genes with multiple poly(A) sites can undergo alternative polyadenylation (APA), producing distinct mRNA isoforms with different 3′ untranslated regions (3′ UTRs) and in some cases different coding regions. Two thirds of all human genes undergo APA. The efficiency of the polyadenylation process regulates gene expression and APA plays an important part in post-transcriptional regulation, as the 3′ UTR contains various cis-elements associated with post-transcriptional regulation, such as target sites for micro-RNAs and RNA-binding proteins.

**Implications of alterations in polyadenylation for endocrine disease:** Alterations in polyadenylation have been found to be causative of neonatal diabetes and IPEX (immune dysfunction, polyendocrinopathy, enteropathy, X-linked) and to be associated with type I and II diabetes, pre-eclampsia, fragile X-associated premature ovarian insufficiency, ectopic Cushing syndrome, and many cancer diseases, including several types of endocrine tumor diseases.

**Perspectives:** Recent developments in high-throughput sequencing have made it possible to characterize polyadenylation genome-wide. Antisense elements inhibiting or enhancing specific poly(A) site usage can induce desired alterations in polyadenylation, and thus hold the promise of new therapeutic approaches.

**Summary:** This review gives a detailed description of alterations in polyadenylation in endocrine disease, an overview of the current literature on polyadenylation and summarizes the clinical implications of the current state of research in this field.

## Introduction

### Background

Almost all eukaryotic pre-messenger RNAs (pre-mRNAs) and several non-coding transcripts have poly(A) sites and are polyadenylated (Shepard et al., 2011; Derti et al., 2012; Lin et al., 2012). Several studies have mapped poly(A) sites genome-wide in humans (Tian et al., 2005; Yan and Marr, 2005; Ozsolak et al., 2010; Fu et al., 2011; Shepard et al., 2011; Derti et al., 2012; Lin et al., 2012). One of the latest found that more than two thirds of all genes have multiple poly(A) sites and can therefore undergo alternative polyadenylation (APA), producing distinct mRNA isoforms with different 3′ untranslated regions (3′ UTRs) (Derti et al., 2012). Polyadenylation and APA are emerging as important regulators of gene expression and alterations in polyadenylation have implications for many diseases (Danckwardt et al., 2008).

This review gives a detailed description of alterations in polyadenylation in endocrine disease, an overview of the current literature on polyadenylation and summarizes the clinical implications of the current state of research in this field. We focus on the co-transcriptional, nuclear polyadenylation, as post-transcriptional and cytoplasmic polyadenylation [alterations to the already added poly(A) tail] is beyond the scope of this review.

### mRNA transcription and polyadenylation

Pre-mRNAs are transcribed from protein-coding genes by the RNA-polymerase II (Pol-II). The nascent pre-mRNA molecule emerging from the Pol-II goes through the co-transcriptional processes of 5′ capping, splicing, and polyadenylation before transcription is terminated. Polyadenylation consists of two steps: (1) Cleavage of the pre-mRNA at a poly(A) site and (2) Addition of an untemplated 3′ poly(A) tail to the upstream cleavage product. The specific cleavage position and the efficiency of the process depend on the interaction between trans-acting polyadenylation factors and cis-elements present in the pre-mRNAs. All eukaryotic pre-mRNAs, except some replication-dependent histone pre-mRNAs, as well as several non-coding transcripts, including micro-RNAs (miRNAs), have poly(A) sites and are polyadenylated (Shepard et al., 2011; Derti et al., 2012; Lin et al., 2012). Polyadenylation is vital for normal mRNA formation, transcription termination and mRNA export from the nucleus, and affects mRNA stability, subcellular localization, and translational efficiency (Sandberg et al., 2008; Ji and Tian, 2009). As polyadenylation is required for transcription termination, the efficiency of the polyadenylation process affects gene expression quantitatively (West and Proudfoot, 2009; Yang et al., 2009; Mapendano et al., 2010).

### Post-transcriptional regulation of gene expression

Tight regulation of gene expression is essential for cells to perform their normal functions and disturbances in gene expression underlie many diseases. Gene expression can be regulated at any step, from the pre-transcriptional epigenetic modifications of the chromatin to the post-translational modifications of the protein product. A quickly responding part of gene expression regulation is the post-transcriptional regulation exerted by RNA-binding proteins (RBPs) and miRNAs. RBPs and miRNAs mainly bind target sequences in the 3′ UTR of mRNAs, thereby controlling mRNA turnover and translation rate (Keene, 2007; Friedman et al., 2009). An example of post-transcriptional regulation is seen in the rapid regulation of insulin level and signaling in response to changes in glucose level. This regulation is exerted through altered stability and translation of insulin mRNA and insulin receptor mRNA, and helps to maintain glucose homeostasis (reviewed in Lee and Gorospe, 2010).

### Role of alternative polyadenylation in post-transcriptional regulation

As RBPs and miRNAs mainly target the 3′ UTR, alterations to the 3′ UTR affect the post-transcriptional regulation. More than two thirds of all mammalian genes encode alternative mRNA isoforms with different 3′ UTRs through APA (Derti et al., 2012). APA therefore plays an important part in the post-transcriptional regulation, as it determines which isoform of the 3′ UTR is expressed (Thomsen et al., 2010). This is seen for the insulin receptor mRNA, from the example above (Levy et al., 1995). Characterization of polyadenylation is therefore essential for understanding both normal cell biology as well as disease. Recent advances in sequencing technologies have made it possible to map poly(A) site usage genome-wide (Mangone et al., 2010; Ozsolak et al., 2010; Fox-Walsh et al., 2011; Fu et al., 2011; Jan et al., 2011; Shepard et al., 2011; Derti et al., 2012; Jenal et al., 2012; Lin et al., 2012; Martin et al., 2012; Pelechano et al., 2012; Yoon et al., 2012; Hoque et al., 2013; Wang et al., 2013; Wilkening et al., 2013). These techniques could be implemented in and improve diagnostics.

## Polyadenylation

### Types of polyadenylation

#### Constitutive polyadenylation

**Figure 1:**
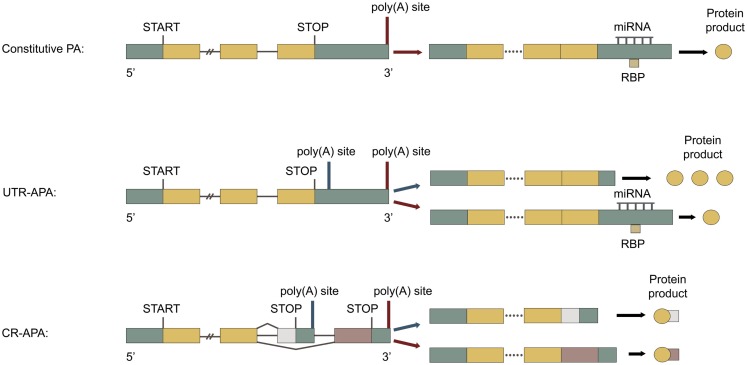
**Types of polyadenylation**. Green boxes represent untranslated regions (UTRs), yellow boxes represent shared exons, pink, and white boxes represent unshared exons and the connecting horizontal lines represent introns. Adapted from Di Giammartino et al. (2011). Top: constitutive polyadenylation: gene contains only one poly(A) site and can therefore not undergo APA. Middle: untranslated region (UTR)-APA: gene contains multiple poly(A) sites located in the 3′ UTR of the terminal exon. APA results in mRNAs with different lengths of 3′ UTR, producing the same protein. Proximal polyadenylation (blue arrow) leads to 3′ UTR shortening, less post-transcriptional regulation, and enhanced protein translation. Bottom: coding region (CR)-APA: gene contains additional poly(A) sites located in the CR of exons and in introns. APA results in mRNAs with different 3′ UTRs and C-terminal CRs, producing distinct protein isoforms. Proximal polyadenylation (blue arrow) produces a mRNA with a different C-terminal CR and 3′ UTR, producing a C-terminally truncated protein isoform.

Genes with a single poly(A) site can only be constitutively polyadenylated (Figure 1, top), as seen for the *INS* gene encoding insulin (Garin et al., 2010). Approximately one third of all human genes only have one poly(A) site (Derti et al., 2012). For these genes the efficiency of polyadenylation regulates gene expression through changes in the transcription termination efficiency (West and Proudfoot, 2009; Yang et al., 2009; Mapendano et al., 2010). Weakened polyadenylation can lead to impaired gene expression and read-through transcription (Higgs et al., 1983), while enhanced polyadenylation can lead to upregulated gene expression (Danckwardt et al., 2004).

#### Alternative polyadenylation

For genes with multiple poly(A) sites, APA can take place (for review of APA see Neilson and Sandberg, 2010; Di Giammartino et al., 2011; Proudfoot, 2011). Only one of the possible poly(A) sites in a pre-mRNA is used for polyadenylation per mRNA transcription event. APA is typically divided into two categories, UTR-APA and coding region (CR)-APA.

##### Untranslated region alternative polyadenylation

Alternative polyadenylation occurring at alternative poly(A) sites located in the 3′ UTR of the last exon is called UTR-APA. It results in mRNAs with the same CR, but with different 3′ UTR length (Figure 1, middle), as seen for the *INSR* gene encoding insulin receptor (Levy et al., 1995). UTR-APA is the most abundant type of APA, accounting for more than half of the APA events (Yan and Marr, 2005; Shepard et al., 2011). About 70% human genes have multiple poly(A) sites in their 3′ UTR and undergo UTR-APA (Derti et al., 2012). The 3′ UTR contains various cis-elements associated with post-transcriptional gene regulation, such as target sites for miRNAs and RBPs, as well as AU-rich elements (AREs) and GU-rich elements (GREs). The 3′ UTR and the factors interacting with it largely determine mRNA stability, subcellular localization, and translational efficiency (Sandberg et al., 2008; Ji and Tian, 2009). It is therefore not surprising, that mutations in the 3′ UTR are associated with many diseases (reviewed in Chen et al., 2006a,b). The general length of the 3′ UTRs has been found to be inversely correlated with cellular proliferation (Sandberg et al., 2008; Elkon et al., 2012). Individual mRNAs with shorter 3′ UTRs are more stable (Mayr and Bartel, 2009; Hogg and Goff, 2010; Yepiskoposyan et al., 2011) and generally produce more protein (Sandberg et al., 2008; Mayr and Bartel, 2009; Singh et al., 2009). In one study 45% of the mRNAs with a general 3′ UTR shortening also had significantly changed expression levels, the majority being upregulated (de Klerk et al., 2012). This would correlate well with a heightened mRNA stability for these transcripts. However, no such significant change in mRNA levels was found in several other studies, where general changes in 3′ UTR length were also seen (Sandberg et al., 2008; Fu et al., 2011; Elkon et al., 2012; Morris et al., 2012). The effects of general changes in 3′ UTR length thus remain unclear. During normal development, escape of miRNA-mediated regulation by APA is seen (Thomsen et al., 2010; Boutet et al., 2012). In addition escape of both miRNA- and RBP-mediated regulation by APA is seen in cancer cells (Boutaud et al., 2003; Sandberg et al., 2008; Mayr and Bartel, 2009). Genes with UTR-APA have common parts of the 3′ UTR expressed in all mRNA isoforms and alternative parts of the 3′ UTR, only expressed in the mRNAs with longer 3′ UTR isoforms. The alternative parts of the 3′ UTR are normally longer, more AU-rich and contain more cis-elements than the common parts (Sandberg et al., 2008; Ji and Tian, 2009). Poly(A) sites preferentially flank cis-elements in the 3′ UTR (Yoon et al., 2012), as seen for miRNA target sites (Ji and Tian, 2009). Interestingly, more than half of the miRNA targets are found downstream of the most proximal poly(A) site in the 3′ UTR (Legendre et al., 2006). The energy expenditure required for synthesizing the longer 3′ UTR isoforms, is used to provide a more refined control of gene expression, through post-transcriptional regulation, as seen for StAR mRNA (Zhao et al., 2005). Surprisingly, the 3′ UTRs of a large number of genes are also expressed alone, separately from their associated protein-coding sequences (Mercer et al., 2011). These expressed 3′ UTRs are suggested both to have trans-acting roles and to function as decoys, which can sequester trans-acting factors, such as miRNAs. UTR-APA in these transcripts would therefore affect these functions, such as generating multiple isoforms of a decoying 3′ UTR, each with different miRNA-binding profiles. For genes undergoing UTR-APA, the efficiency of polyadenylation contributes to gene expression through the transcription termination efficiency. In addition to this the choice of different poly(A) sites through APA plays an important part in post-transcriptional regulation, as it defines which isoform of the 3′ UTR is expressed (Thomsen et al., 2010). Dysfunctional polyadenylation can lead to changes in the APA pattern, altering the 3′ UTR, and to changes in polyadenylation efficiency, both affecting gene expression.

##### Coding region alternative polyadenylation

Alternative polyadenylation occurring at alternative poly(A) sites located in introns or in the CR of exons is called CR-APA. It results in mRNAs with both different C-terminal CRs and 3′ UTRs (Figure 1, bottom), as seen for the *CALCA* gene encoding calcitonin/CGRP (Amara et al., 1984; Zhou et al., 2007). CR-APA often occurs together with alternative splicing of the last exon and it is suggested that there is a dynamic competition between splicing and polyadenylation (Tian et al., 2007; Evsyukova et al., 2012). About 20% of human genes have at least one utilized intronic poly(A) site, usually located in large introns with weak 5′ splice sites (Tian et al., 2007). Thousands of dormant intronic poly(A) sites have been characterized, which are normally suppressed and therefore not utilized (Yao et al., 2012a). Enhanced usage of intronic poly(A) sites is associated with proliferation (Elkon et al., 2012) and with a general transcriptional upregulation (Berg et al., 2012). Polyadenylation within the CR of an exon can convert a Tyr codon to a stop codon and thereby give rise to a functional mRNA (Yao et al., 2012b). Unlike mRNAs with acquired premature termination codons, which also generate C-terminal truncated proteins, the mRNAs generated by CR-APA are not targeted by nonsense-mediated decay (Lejeune and Maquat, 2005). For genes undergoing CR-APA, the efficiency of polyadenylation contributes to gene expression through the transcription termination efficiency. Additionally, the choice of different poly(A) sites through APA determines both the mRNA coding potential and 3′ UTR isoform. Dysfunctional polyadenylation can lead to changes in the APA pattern, altering the protein product and 3′ UTR, and to changes in polyadenylation efficiency, both affecting gene expression.

### Cis-elements and the trans-acting polyadenylation factors

#### Cis-elements guiding polyadenylation

**Figure 2:**
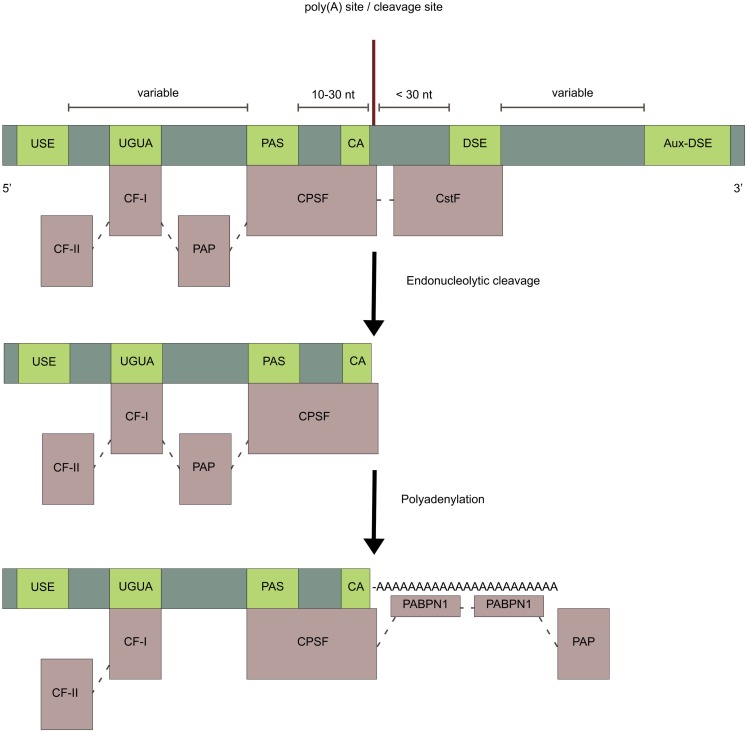
**Cis-elements and polyadenylation factors**. Light-green boxes represent cis-elements in the pre-mRNA and light-purple boxes represent polyadenylation factors. The endonucleolytic cleavage of the pre-mRNA is made by the CPSF-subunit CPSF-73 at the poly(A) site, typically immediately 3′ of a CA dinucleotide. After cleavage, an untemplated poly(A) tail of about 250 adenosine-nucleotides, is added to the upstream cleavage product by PAP. This is stimulated by the CPSF through the nuclear poly(A)-binding protein (PABPN1) which binds the growing poly(A) tail and controls its length.

The cis-elements guiding polyadenylation of the nascent pre-mRNA are positioned upstream or downstream relative to a poly(A) site (Figure 2) (reviewed in Tian and Graber, 2012). The poly(A) site is where the pre-mRNA molecule is cleaved, usually immediately 3′ of a CA dinucleotide, and where the poly(A) tail on the mature mRNA starts. Poly(A) sites normally contain two core cis-elements: (1) The polyadenylation signal (PAS) placed 10–30nt upstream of the poly(A) site, consisting of the canonical sequence element, AAUAAA, found in more than half of mammalian genes, or a weaker non-canonical variant (Beaudoing et al., 2000; Zhang et al., 2005; Ho and Gunderson, 2011; Wang et al., 2013). (2) The U/GU-rich downstream sequence element (DSE), located up to 30nt downstream of the poly(A) site. Additional elements such as U-rich upstream sequence elements (USE), located just upstream of the PAS (Moreira et al., 1998), G-rich auxiliary downstream elements (Aux-DSE) (Chen and Wilusz, 1998), located downstream of the DSE, and UGUA motifs (Brown and Gilmartin, 2003; Venkataraman et al., 2005; Yang et al., 2011) are found around some poly(A) sites and all act as enhancers of polyadenylation. The strength of a poly(A) site depends on the exact sequence of its surrounding cis-elements, as well as their spatial placement, which together determinates the affinity for the trans-acting factors. In genes with only one poly(A) site, the PAS is typically canonical (Beaudoing et al., 2000). In genes with multiple poly(A) sites, the most distal poly(A) site generally has a canonical PAS whereas the proximal poly(A) sites generally have a non-canonical PAS (Beaudoing et al., 2000; Tian et al., 2005, 2007; Shepard et al., 2011; de Klerk et al., 2012; Lin et al., 2012; Yoon et al., 2012; Wang et al., 2013). The proximal poly(A) sites of skipped terminal exons however tend to be associated with a canonical PAS as well (Tian et al., 2007). Poly(A) sites with non-canonical cis-elements are weaker and require additional factors for efficient utilization (Fu et al., 2011), as seen for the weak proximal poly(A) site in the *CALCA* gene encoding calcitonin/CGRP (Van Oers et al., 1994; Lou et al., 1998). The distal poly(A) sites with canonical cis-elements work as a secure last option for polyadenylation, ensuring that transcription is terminated. This minimizes potentially damaging read-through transcription into downstream genes, which normally occurs for a small proportion of transcripts (Dresser et al., 1995). The arrangement of the cis-elements with one A-rich element associated with one or more U-rich elements around the poly(A) site is generally conserved across eukaryotes (Millevoi and Vagner, 2010). A comparison of orthologous human, rhesus, dog, mouse, and rat genes, showed that poly(A) site usage is strikingly similar in orthologous tissues between species (Tian et al., 2005; Derti et al., 2012). The canonical PASs are positionally conserved between species (Derti et al., 2012), opposed to the non-canonical PASs, which have been found to be less conserved (Ara et al., 2006). Intronic poly(A) sites are also less conserved than poly(A) sites in the 3′ UTR of terminal exons (Tian et al., 2007). The non-canonical proximal poly(A) sites tend to have more conserved flanking regions (Wang et al., 2008), in which conserved motifs have been found, possibly explaining the relative frequent usage of these poly(A) sites (Nunes et al., 2010; Ozsolak et al., 2010).

#### Trans-acting polyadenylation factors (the human 3 ′-end processing complex)

The human 3′-end processing complex consists of more than 80 proteins (Shi et al., 2009), which are highly conserved between species (Darmon and Lutz, 2012). An important part of this complex, the core polyadenylation machinery, consists of five multi-subunit protein factors: cleavage and polyadenylation specificity factor (CPSF), cleavage stimulation factor (CstF), cleavage factor I (CF-I), cleavage factor II (CF-II), and poly(A) polymerase (PAP) (Table 1; Figure 2) (reviewed in Chan et al., 2011). The endonucleolytic cleavage of the pre-mRNA is made by the CPSF-73 subunit at the poly(A) site (Figure 2) (Mandel et al., 2006). The pre-mRNA is typically cleaved immediately 3′ of a CA dinucleotide (Danckwardt et al., 2008), although the exact cleavage site is usually clustered within a few nucleotides (Beaudoing et al., 2000; Pauws et al., 2001; Tian et al., 2005; Lin et al., 2012). After cleavage an untemplated poly(A) tail of about 250 adenosine-nucleotides is added to the upstream cleavage product by PAP (Figure 2). This is stimulated by the CPSF through the nuclear poly(A)-binding protein (PABPN1), which binds the growing poly(A) tail and controls its length (Kühn et al., 2009). Many of the other proteins in the human 3′-end processing complex do not play a direct role in the 3′-end formation, but instead couple polyadenylation to other cellular processes.

**Table 1:** **The core polyadenylation machinery**.

Factor	Subunits	Necsessary for	Interactions with other core factors	Target cis-element	Reference
CPSF	CPSF-30, CPSF-73, CPSF-100, CPSF-160, hFip1, Wdr33, and symplekin	Cleavage step and polyadenylation step	CstF, CF-II, and PAP	The PAS (AAUAAA), binding mediated by CPSF-160	Murthy and Manley (1995), Chan et al. (2011)
CstF	CstF-50, CstF-64, and CstF-77	Cleavage step	CPSF and CF-II	The DSE (U/GU-rich), binding mediated by CstF-64	MacDonald et al. (1994), Chan et al. (2011)
CF-I	CF-I-25 and either CF-I-68 or CF-I-59	Cleavage step	CF-II and PAP	The auxiliary motif UGUA	Venkataraman et al. (2005), Chan et al. (2011)
CF-II	hPcf11 and hClp1	Cleavage step	CPSF, CstF, and CF-I	None	Chan et al. (2011)
PAP	None	Cleavage step and polyadenylation step	CPSF and CF-I	None	Chan et al. (2011)

### Regulation of polyadenylation

Polyadenylation is influenced by three factors: (1) The strength of the cis-elements. (2) The concentration and activity of polyadenylation factors. (3) The concentration and activity regulatory factors (Barabino and Keller, 1999). These determine the efficiency of polyadenylation and the pattern of APA. The regulation of constitutive and alternative polydenylation differs and even CR-APA and UTR-APA are regulated differently. This differentiated regulation of APA is seen during T-cell activation, where CR-APA occurs at both early and late stages of activation, utilizing both more proximal and more distal poly(A) sites. In contrast, UTR-APA is most evident in late stages of activation and dominantly utilize more proximal poly(A) sites (Sandberg et al., 2008). The differentiated regulation of APA is also seen during stem cell differentiation where UTR-APA generally switches toward utilizing more distal poly(A) sites, opposed to CR-APA which switches toward utilizing both more proximal and more distal poly(A) sites (Shepard et al., 2011). CR-APA often occurs together with alternative splicing of the last exon and it is suggested that there is a dynamic competition between splicing and polyadenylation (Tian et al., 2007; Evsyukova et al., 2012). CR-APA is thus both regulated by polyadenylation and splicing. The regulation of polyadenylation is complex and the involved factors interact with several other cellular processes. Part of the regulation is mediated through mechanistic interactions with polyadenylation factors, enhancing, or inhibiting their function, competing for the binding to their cis-elements or reducing their free levels in the nucleus (reviewed in Millevoi and Vagner, 2010).

#### Regulation by levels and variants of polyadenylation factors

**Figure 3:**
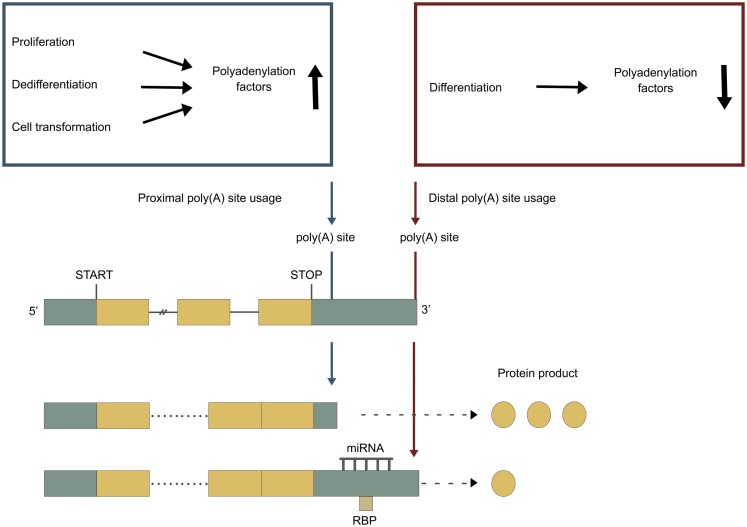
**APA under different cellular conditions**. Green boxes represent untranslated regions (UTRs), yellow boxes represent exons, and the connecting horizontal lines represent introns. Adapted from Di Giammartino et al. (2011). Blue poly(A) site represents a proximal poly(A) site, which is normally non-canonical and weaker. Red poly(A) site represents a distal poly(A) site, which is normally canonical and stronger. Blue box: proliferation, dedifferentiation and cell transformation upregulates the levels of polyadenylation factors, enhancing the utilization of the weaker proximal poly(A) site. Red box: differentiation downregulates the levels of polyadenylation factors, leading to a utilization of the stronger distal poly(A) site.

It is hypothesized that higher levels of polyadenylation factors lead to improved utilization of weaker proximal poly(A) sites, encountered earlier during transcription (Figure 3). This is seen during proliferation, where upregulated levels of polyadenylation factors correlate with proximal poly(A) site usage (Elkon et al., 2012). It is also seen for many of the individual core polyadenylation factors (see sections below). Every component, but not every subunit, of the core polyadenylation machinery are regulated by post-translational modifications, such as methylation, sumoylation, acetylation, and phosphorylation (reviewed in Ryan and Bauer, 2008).

##### Cleavage and polyadenylation specificity factor

During colorectal carcinoma progression and in breast cancer cell lines, upregulated levels of CPSF-subunits CPSF-73, CPSF-160, CPSF-30, and symplekin correlate with proximal poly(A) site usage (Mayr and Bartel, 2009; Morris et al., 2012). Interestingly, symplekin is also highly overexpressed in colon, lung, muscle, and prostate tumors where it promotes tumorigenesis (Buchert et al., 2010). CPSF-160 and symplekin are expressed in high levels in germ cells where the general 3′ UTR length is the shortest (Dass et al., 2001; Liu et al., 2007). CPSF-100 was conversely downregulated. During the induction of stem cells from somatic cells, all CPSF-subunits except CPSF-100 and hFip1, are upregulated. This correlates with a general shift toward proximal poly(A) site usage. During embryonic development all CPSF-subunits except hFip1 are downregulated. This correlates with a general shift toward distal poly(A) site usage (Ji and Tian, 2009; Ji et al., 2009). The examples indicate that upregulated levels of CPSF enhance polyadenylation, leading to proximal poly(A) site usage. Interestingly, knockdown of the CPSF-subunit hFip1 increases DNA damage and chromosome breakage (Stirling et al., 2012). This is properly caused by dysfunctional polyadenylation, leading to defective transcription termination, read-through transcription and formation of R-loops (see section *Sequence changes in genes encoding polyadenylation factors*, page 15).

##### Cleavage stimulation factor

Upregulated levels of the CstF subunit CstF-64 correlate with proximal poly(A) site usage, during colorectal carcinoma progression and in breast cancer cell lines (Mayr and Bartel, 2009; Morris et al., 2012). Upregulated levels of CstF-64 enhance utilization of proximal poly(A) sites in the mouse Testis Brain RNA-Binding Protein (*TB-RBP*) gene in male germ cells (Chennathukuzhi et al., 2001). CstF-64 is also upregulated in activated immune cells, enhancing the usage of proximal poly(A) sites (Shell et al., 2005; Sandberg et al., 2008). All CstF subunits are upregulated, during the induction of stem cells from somatic cells, correlating with a general shift toward proximal poly(A) site usage. Additionally, these subunits are downregulated during embryonic development, correlating with a general shift toward distal poly(A) site usage (Ji and Tian, 2009; Ji et al., 2009). When comparing the used poly(A) sites in genes that lengthen and genes that shorten during the induction of stem cells from somatic cells, there was major differences in the DSE. The DSE is where CstF binds, indicating that CstF plays a crucial role in the regulation of poly(A) site usage (Ji and Tian, 2009). The CstF-64 ortholog τCstF-64, expressed at highest levels in testis and brain, is necessary for spermatogenesis and fertilization (Dass et al., 2007; Hockert et al., 2011). Both CstF-64 and τCstF-64 are expressed in high levels in germ cells, which have the shortest general 3′ UTR length (Dass et al., 2001; Liu et al., 2007). Loss of τCstF-64 changes gene expression genome-wide in germ cells, leads to more frequent usage of distal poly(A) sites and causes more read-through transcription due to aberrant transcription termination (Li et al., 2012c). RNAi-mediated depletion of CstF-64 has a small effect on APA, but leads to upregulation of τCstF-64, suggesting that CstF-64 and τCstF-64 play redundant roles (Yao et al., 2012a). In support of this, during differentiation of C2C12 myoblasts τCstF-64 is also upregulated at the same time as the three subunits of CstF are downreulated (Ji et al., 2009). Codepletion of CstF-64 and τCstF-64 leads to significant changes in APA, with enhanced usage of distal poly(A) sites (Yao et al., 2012a). Together this indicates that upregulation of CstF enhances polyadenylation, leading to the usage of proximal poly(A) sites. A CstF-64 splice variant βCstF-64 is expressed specifically in nervous tissue and is thought to have regulatory functions here (Shankarling et al., 2009). Interestingly, CstF-64 increases fivefold during cell cycle, at G_0_ to S phase transition (Martincic et al., 1998) and low levels of CstF-64 cause reversible cell cycle arrest while depletion causes apoptosis (Takagaki and Manley, 1998).

##### Cleavage factor I

Cleavage factor I has been found to be important for non-canonical poly(A) site usage by enhancing the recruitment of CPSF (Brown and Gilmartin, 2003; Venkataraman et al., 2005; Sartini et al., 2008; Yang et al., 2011). In the germ cells, where the general 3′ UTR length is the shortest, the two CF-I subunits CF-I-25 and CF-I-68 are upregulated (Liu et al., 2007; Sartini et al., 2008). These two subunits are also upregulated during the induction of stem cells from somatic cells, correlating with a general shift toward proximal poly(A) site usage (Ji and Tian, 2009). These examples correlate well with the proposed function of CF-I in promoting non-canonical poly(A) site usage. However, reduced levels of CF-I has been found to enhance more proximal poly(A) site usage (Kubo et al., 2006; Kim et al., 2010). Loss of function of CF-I-25 and CF-I-68, but not of CF-I-59, have also been found to enhance genome-wide proximal poly(A) site usage (Gruber et al., 2012; Martin et al., 2012). This indicates that CF-I has other functions than enhancing the recruitment of CPSF, yet to be fully explained. Interestingly, downregulation of CF-I-68 has also been found to increase tumor invasiveness (Yu et al., 2008).

##### Cleavage factor II

The CF-II subunit hClp1 is upregulated during the induction of stem cells from somatic cells, correlating with a general shift toward proximal poly(A) site usage (Ji and Tian, 2009). Likewise, hClp1 is downregulated during embryonic development, correlating with a general shift toward distal poly(A) site usage (Ji and Tian, 2009; Ji et al., 2009). In the germ cells, where there is a short general 3′ UTR length, hClp1 is also upregulated (Liu et al., 2007). Low levels of the CF-II subunit hPcf11 reduces transcription termination efficiency, due to weakened polyadenylation (West and Proudfoot, 2008). Taken together it shows that upregulation of CF-II enhances polyadenylation, leading to proximal poly(A) site usage.

##### Poly(A) polymerase

In breast cancer cell lines, upregulated levels of PAP correlate with proximal poly(A) site usage (Mayr and Bartel, 2009). PAP is also upregulated during the induction of stem cells from somatic cells, correlating with a general shift toward proximal poly(A) site usage (Ji and Tian, 2009). These examples indicate that upregulation of PAP enhances polyadenylation, leading to usage of proximal poly(A) sites. PAP is post-translationally modified during cell cycle progression, with the modifications reflecting the proliferative state of the cell (Thomadaki et al., 2008). PAP modifications also correlate with apoptosis (Thomadaki et al., 2008). One of the PAP regulators, poly(ADP-ribose) polymerase 1 (PARP1), modifies PAP during heat shock, leading to an inhibition of polyadenylation (Di Giammartino et al., 2013). In addition to PAP, three nuclear non-canonical PAPs exists: Neo-PAP, TRAP, and Star-PAP. Neo-PAP has a function equal to that of PAP (Topalian et al., 2001). TRAP is only expressed in testis and has functions not fully understood (Lee et al., 2000). Star-PAP is necessary for the 3′-end formation of selected mRNAs, such as the cytoprotective enzyme heme oxygenase-1, which is upregulated in response to oxidative stress (Mellman et al., 2008) and the BIK protein, an initiator of mitochondrial apoptosis (Li et al., 2012b). Star-PAP activity is modulated directly by phosphatidylinositol 4,5-bisphosphate (PI4,5P_2_) and Star-PAP associates with both PIPKIα, which can generate new PI4,5P_2_, and with the PI4,5P_2_ sensitive protein kinase CKIα, which can phosphorylate Star-PAP (Laishram et al., 2011). The phosphorylation of Star-PAP is critical for its activity and PI4,5P_2_ sensitivity.

##### Other polyadenylation factors

The nuclear poly(A)-binding protein (PABPN1) is upregulated during the induction of stem cells from somatic cells, correlating with a general shift toward proximal poly(A) site usage (Ji and Tian, 2009). However, PABPN1 recently has been shown to suppress polyadenylation at weaker proximal poly(A) sites and loss of PABPN1 results in genome-wide 3′ UTR shortening (de Klerk et al., 2012; Jenal et al., 2012). Loss of PABPN1 also increases apoptotic markers in affected cells (Bhattacharjee and Bag, 2012).

#### Regulation by non-polyadenylation factors

##### Splice factors

Many known splice factors have been shown to affect polyadenylation (Table 2).

**Table 2:** **Splice factors affecting polyadenylation**.

Splice factor	Motif	Mechanism	Effect on polyadenylation	Reference
FOX-1/FOX-2 (neuronal-specific)	UGCAUG	Motifs are found in 3′ UTRs between poly(A) sites, indicating a role in APA regulation	Unknown	Wang et al. (2008), Zhou et al. (2007)
Nova (neuronal-specific)	YCAY	Enhances or inhibits polyadenylation depending on the position of its motif relative to the PAS	Enhance/inhibit	Licatalosi et al. (2008)
U1	5′ splice site	Represses adjacent downstream intronic poly(A) site usage	Inhibit	Kaida et al. (2010), Berg et al. (2012), Vorlová et al. (2011), Vickers et al. (2011)
		Inhibits the PAP	Inhibit	Gunderson et al. (1998)
	Intronic enhancer element (with both 5’ and 3’ splice sites)	Enhances upstream polyadenylation	Enhance	Lou et al. (1998)
U1A (component of U1)	AUGCN(1-3)C	Binds motifs downstream of poly(A) site and inhibits CstF binding to the DSE	Inhibit	Phillips et al. (2004)
	USE	Enhances polyadenylation	Enhance	Hall-Pogar et al. (2007)
	AUUGCAC	Inhibits the PAP	Inhibit	Gunderson et al. (1997)
U2	3′ splice site	Enhances adjacent downstream poly(A) site usage	Enhance	Kyburz et al. (2006)
U2 auxiliary factor 1 (U2AF1)	USE	Enhances polyadenylation	Enhance	Danckwardt et al. (2007)
U2 auxiliary factor 2 (U2AF2)	3′ splice site	Enhances adjacent downstream poly(A) site usage	Enhance	Millevoi et al. (2006)
		Inhibits the PAP	Inhibit	Ko and Gunderson (2002)
hnRNP F	DSE	Competes with CstF for DSE binding	Inhibit	Veraldi et al. (2001)
hnRNP L	CA-rich elements	Inhibits the usage of intronic poly(A) sites	Inhibit	Hung et al. (2008)
hnRNP H	Aux-DSE	Enhances polyadenylaiton	Enhance	Dalziel et al. (2007)
hnRNP I (PTB)	DSE	Competes with CstF for DSE binding	Inhibit	Castelo-Branco et al. (2004)
	USE	Enhances polyadenylation	Enhance	Millevoi et al. (2009), Hall-Pogar et al. (2007), Blechingberg et al. (2007)
PTB associated splicing factor	USE	Enhances polyadenylation	Enhance	Hall-Pogar et al. (2007)
SR proteins	Unknown	Enhances polyadenylation	Enhance	Blechingberg et al. (2007)
SRp20	Intronic enhancer element (with both 5’ and 3’ splice sites)	Enhances upstream polyadenylation	Enhance	Lou et al. (1998)
SRp75	Unknown	Inhibits the PAP	Inhibit	Ko and Gunderson (2002)
SRm160	Unknown	Enhances polyadenylation	Enhance	McCracken et al. (2002, 2003)

##### Non-splice factors

Many non-splice factors have also been shown to affect polyadenylation (Table 3).

**Table 3:** **Non-splice factors affecting polyadenylation**.

Factor	Motif	Mechanism	Effect on polyadenylation	Reference
E2F	TTGGCGG	Motifs are enriched in promoter-regions of many polyadenylation factors and E2F enhances their expression	Upregulation of polyadenylation factors leading to 3′ UTR shortening	Elkon et al. (2012), Ji and Tian (2009)
Hu proteins	U-rich sequences	Inhibits CstF binding to the DSE at poly(A) sites containing U-rich sequences	Inhibit	Zhu et al. (2007)
NS1 (Influenza A virus protein)	None	Interacts with CPSF-30 and inhibits polyadenylation of host pre-mRNAs in infected cells	Inhibit	Nemeroff et al. (1998)
14-3-3ε	None	Inhibits the PAP and redistributes it to the cytoplasm	Inhibit	Kim et al. (2003)
CSR1	None	Redistributes CPSF-73 to the cytoplasm	Inhibit	Zhu et al. (2009)
IRBIT	None	In response to oxidative stress, IRBIT is phosphorylated, whereby it both inhibits PAP and redistributes the CPSF-subunit hFip1 to the cytoplasm	Inhibit	Kiefer et al. (2009)
p53	None	In response to DNA damage, p53 complexes with BARD1 and CstF-50, lowering the free levels of CstF-50	Inhibit	Nazeer et al. (2011)
BRCA1	None	In response to DNA damage, BRCA1 complexes with BARD1 and CstF-50, lowering the free levels of CstF-50	Inhibit	Kleiman and Manley (2001)
Cdc73	None	Associates with CPSF-CstF complexes and stimulates polyadenylation of specific target genes	Enhance	Rozenblatt-Rosen et al. (2009)
HSF1	None	In response to cellular stress, HSF1 complexes with symplekin and CstF-64 and enhances polyadenylation of Hsp70	Enhance	Xing et al. (2004)
P54	USE	Enhances polyadenylation	Enhance	Hall-Pogar et al. (2007)
p38 MAPK	None	In response to cellular stess, p38 MAPK phosphorylates RBPs blocking the USE, leading to their dissociation, and upregulates several polyadenylation factors, both events leading to enhanced polyadenylation	Enhance	Danckwardt et al. (2011)
T3	None	T3 stimulates the nuclear polyadenylaiton step, mediated by PAP, increasing poly(A) tail length of P450R	Enhance	Liu and Waxman (2002)
FSH	None	Stimulates proximal poly(A) site usage in *CREM* gene	Enhance	Foulkes et al. (1993)
Estrogen	None	Induces 3′ UTR shortening of the DNA replication regulator CDC6	Enhance	Akman et al. (2012)

#### Regulation by transcription and co-transcriptional processes

##### Transcription

Polyadenylation is necessary for transcription termination (West and Proudfoot, 2009). It also affects transcription re-initiation at upstream promoters (Mapendano et al., 2010). The CTD of the Pol-II is necessary for the cleavage step in mammals (Licatalosi et al., 2002). It recruits CPSF, CstF, and CF-II and transfers them to their cis-elements in the pre-mRNA, as it emerges from the Pol-II (McCracken et al., 1997; Lunde et al., 2010). The recruitment is mediated by phosphorylation of the Pol-II CTD serine 2 (Ser2) residues, which are progressively phosphorylated during Pol-II elongation (Ahn et al., 2004). Poly(A) sites located near the 5′-end of genes are normally not used, preventing premature 3′-end processing. This is probably because of the low Ser2 phosphorylation in the Pol-II CTD right after transcription initiation, resulting in inefficient recruitment of polyadenylation factors (Guo et al., 2011). The general transcription factor TFIIB is required for recruiting CPSF and CstF to the promoter (Wang et al., 2010). The transcription elongation rate affects the choice of poly(A) site, with a lower rate enhancing usage of proximal poly(A) sites (Pinto et al., 2011). Transcription elongation factor ELL2 also enhances polyadenylation (Martincic et al., 2009). Pol-II occupancy is found to be higher at distal poly(A) sites than at proximal poly(A) sites (Lin et al., 2012). Transcriptional activity regulates APA, with highly expressed genes expressing short 3′ UTR isoforms and lowly expressed genes expressing long 3′ UTR isoforms. This as the Pol-II is more likely to pause at poly(A) sites in the highly expressed genes (Ji et al., 2011). As the mRNAs with short 3′ UTRs generated from the highly expressed genes are more stable and produce more protein, this effect potentiates the gene expression difference. Transcriptional activators have also been shown to enhance polyadenylation (Nagaike et al., 2011). The choice of alternative 5′ UTRs and promoters affect the choice of poly(A) site and thus APA (Winter et al., 2007; Ji et al., 2011).

##### Capping

The presence of a 5′-end m7GpppG cap has been shown to positively affect the efficiency of polyadenylation (Cooke and Alwine, 1996). The nuclear cap-binding complex (CBC), bound to the 5′ cap, is necessary for assembling a stable 3′-end processing complex. Depletion of CBC thus strongly reduces the efficiency of the cleavage step (Flaherty et al., 1997).

##### Splicing

The patterns of APA and alternative splicing correlate across tissues, indicating coordinated regulation of these processes (Wang et al., 2008). Because the used intronic poly(A) sites are usually located in large introns with weak 5′ splice sites, which require more time to be spliced out, it has been suggested that there is a dynamic competition between splicing and polyadenylation (Tian et al., 2007; Evsyukova et al., 2012). Many of the trans-factors involved in polyadenylation are also known to take part in splicing (Shi et al., 2009; Evsyukova et al., 2012). Similarly, a number of known splice factors have also been shown to affect polyadenylation (Table 2).

#### Regulation by other processes

##### RNA structure

The secondary structure of RNA can affect polyadenylation. This is seen in HIV-1-strains where the proximal poly(A) site dominantly fold into a hairpin structure, thereby inhibiting polyadenylation (Gee et al., 2006). Highly used poly(A) sites are positively associated with an energetically favorable mRNA structure near poly(A) sites that exposes the PAS (Khaladkar et al., 2011).

##### Nucleosome positioning

Decreased nucleosome density is found around poly(A) sites, most significantly around the actively used ones (Spies et al., 2009). Increased nucleosome density is found downstream of poly(A) sites, most significantly downstream of the actively used ones (Spies et al., 2009; Khaladkar et al., 2011). Highly expressed genes have a lower nucleosome density around poly(A) sites than genes expressed at low levels (Ji et al., 2011).

##### Epigenetic modifications

Allele-specific poly(A) site usage is seen in an imprinted mouse gene (*H13*), as a result of DNA-methylation. A CpG island separates polyA sites utilized in *H13*. Alleles without methylation of the CpG island utilize a proximal poly(A) site, generating a truncated H13, whereas alleles with methylation of the CpG island, utilize downstream poly(A) sites (Wood et al., 2008). Histone methylation (H3K36me3) decreases downstream of used poly(A) sites (Lian et al., 2008; Wang et al., 2013). Highly expressed genes have increased levels of histone methylation (H3K4me3 and H3K36me3) around proximal poly(A) sites compared to around distal poly(A) sites (Ji et al., 2011; Lin et al., 2012). The pattern of histone modifications can thus characterize poly(A) sites and even discriminate between poly(A) sites with high and low usage, suggesting a link between chromatin structure and mRNA structure (Khaladkar et al., 2011).

##### Circadian rhythms

The suppressor of cytokine signaling 3 (SOCS3) mRNA, encodes a protein important for the leptin signaling pathway. SOCS3 mRNA is expressed in two isoforms due to APA. The two isoforms are expressed in an oscillating pattern and in opposite phases (Ptitsyn and Gimble, 2007).

## Methods for Characterizing Poly(A) Site Usage

### Complementary DNAs, expressed sequence tags, and microarrays

Early studies to identify poly(A) sites in human were done by aligning complementary DNAs (cDNAs) and expressed sequence tags (ESTs) to the genome, thereby identifying the 3′ end of genes. These analyses revealed that more than half of human genes have multiple poly(A) sites and undergo APA (Beaudoing et al., 2000; Tian et al., 2005; Yan and Marr, 2005). Later studies using microarray platforms were designed to characterize APA in different cell types, cancers, and at different development stages. These analyses showed that a general 3′ UTR shortening associates with increased proliferation, dedifferentiation, and cell transformation (Sandberg et al., 2008; Ji and Tian, 2009; Singh et al., 2009) whereas a general 3′ UTR lengthening associates with cell differentiation (Ji and Tian, 2009; Ji et al., 2009). Poly(A) site usage were also found to differ according to tissue type, developmental stage, genotype, and cancer subtype (Kwan et al., 2008; Sandberg et al., 2008; Ji and Tian, 2009; Ji et al., 2009; Singh et al., 2009). The cDNA and EST methods have limited statistical power and microarrays are limited to comparing the results only with already known poly(A) sites and have difficulties with quantifying the different 3′-UTR isoforms produced by APA. Thus new methods for characterizing poly(A) site usage were needed.

### High-throughput sequencing techniques

With the development of new high-throughput sequencing techniques, several methods have been developed to specifically characterize poly(A) site usage genome-wide (Mangone et al., 2010; Ozsolak et al., 2010; Fox-Walsh et al., 2011; Fu et al., 2011; Jan et al., 2011; Shepard et al., 2011; Derti et al., 2012; Jenal et al., 2012; Lin et al., 2012; Martin et al., 2012; Pelechano et al., 2012; Yoon et al., 2012; Hoque et al., 2013; Wang et al., 2013; Wilkening et al., 2013). These new techniques provide higher coverage than any of the previous methods, allow better detection of the poly(A) sites and give a more precise quantification of their relative usage. High-throughput sequencing studies have confirmed that more than half of human genes have multiple poly(A) sites and undergo APA (Derti et al., 2012). They have also confirmed that 3′ UTR shortening associates with proliferation (Elkon et al., 2012) and that 3′ UTR lengthening associates with differentiation (Shepard et al., 2011). However, they have not unanimously confirmed that 3′ UTR shortening associates with cell transformation, but instead found that APA patterns can shift for both more proximal and more distal poly(A) site usage during cell transformation (Fu et al., 2011; Elkon et al., 2012; Lin et al., 2012; Morris et al., 2012). Several computational studies have been performed to characterize patterns in the cis-elements around used poly(A) sites obtained from experiments (Beaudoing et al., 2000; Legendre and Gautheret, 2003; Tian et al., 2005; Ara et al., 2006; Nunes et al., 2010; Ozsolak et al., 2010). Computational methods have also been developed to identify poly(A) sites (Tabaska and Zhang, 1999; Graber et al., 2002; Hajarnavis et al., 2004; Bajic et al., 2005; Cheng et al., 2006; Retelska et al., 2006; Akhtar et al., 2010).

## The Polyadenylation Pattern

### Changed patterns of polyadenylation under different cellular conditions

**Figure 4:**
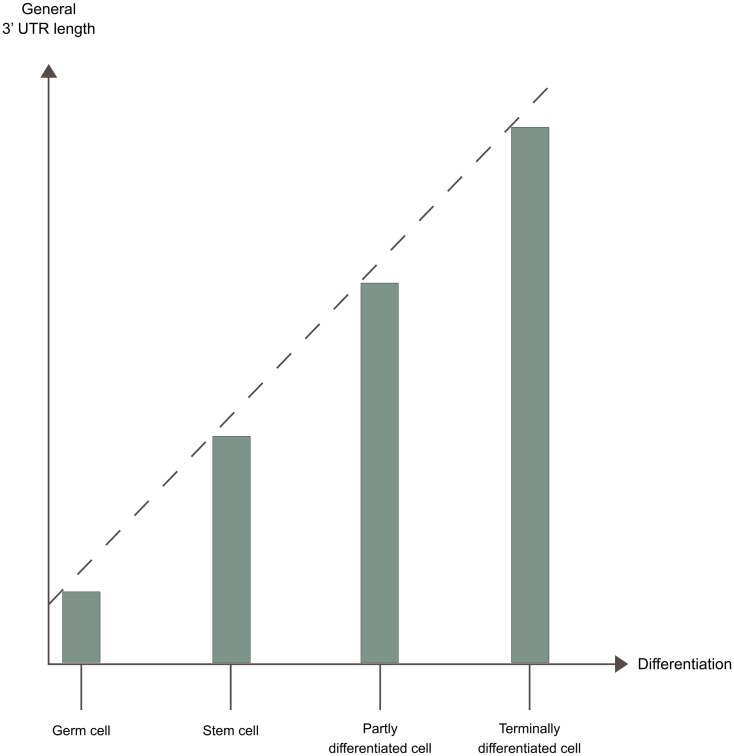
**3′ UTR length and developmental potential**. The developmental potency of a cell seems to be inversely correlated to 3′ UTR length. The 3′ UTR is shortest in germ cells < stem cells < partly differentiated cells < terminally differentiated cells, due to different APA patterns in these cells.

Changes in cellular conditions can cause a widespread dynamic shift in the use of poly(A) sites by APA, altering the 3′ UTRs, and in some cases the coding potential of the mRNAs. A shift toward more proximal poly(A) site usage associates with increased proliferation (Sandberg et al., 2008; Elkon et al., 2012) and with dedifferentiation (Ji and Tian, 2009). In contrast, shift toward more distal poly(A) site usage associates with cell differentiation (Figure 3) (Ji and Tian, 2009; Ji et al., 2009; Mangone et al., 2010; Hilgers et al., 2011; Shepard et al., 2011). The developmental potency of a cell seems to be inversely correlated to 3′ UTR length, with the 3′ UTR being shortest in germ cells < stem cells < partly differentiated cells < terminally differentiated cells (Figure 4) (Ji and Tian, 2009; Shepard et al., 2011). 3′ UTR shortening has also been reported to be associated with cell transformation (Mayr and Bartel, 2009; Singh et al., 2009; Lin et al., 2012; Morris et al., 2012). Recent studies have however found that this association is inconsistent (Fu et al., 2011; Elkon et al., 2012). Interestingly, there is an enrichment of binding sites for several transcription factors involved in proliferation/differentiation, including E2F, c-myc, and p53 in the promoter-regions of many of the genes coding for polyadenylation factors (Ji and Tian, 2009; Elkon et al., 2012). High levels of E2F have been shown to enhance the expression of polyadenylation factors. E2F transcription factors are upregulated during proliferation (Elkon et al., 2012). This might explain the shift in the APA during proliferation, while changes in levels of other transcription factors might explain the shift in APA during differentiation. Activation of neurons also induce 3′ UTR shortening for multiple genes (Flavell et al., 2008). This is due to the relative shortage of splice factor U1 generated by the transcriptional upregulation found in activated neurons, as shortage of U1 leads to enhanced usage of proximal poly(A) sites (Berg et al., 2012).

#### During cell differentiation/dedifferentiation

Generally the 3′ UTRs shorten when inducing pluripotent stem cells from somatic cell types, the opposite of what happens during embryonic development. When inducing from germ cells 3′ UTRs generally lengthen, the opposite of what happens during postnatal testis development (Ji and Tian, 2009). The genes with 3′ UTR shortening during the induction were more likely to have had 3′ UTR lengthening during embryonic development. Vice versa, the genes with 3′ UTR lengthening during the induction were more likely to have had 3′ UTR shortening during embryonic development (Ji and Tian, 2009). The proximal poly(A) sites highly responsive to cell state change during the induction of somatic cells, were also highly regulated during embryonic development, but in the opposite direction. When comparing these poly(A) sites with less responsive proximal poly(A) sites in other genes, they were more conserved, had stronger DSE and lead to longer alternative 3′ UTRs (Ji and Tian, 2009). A recent high-throughput sequencing study also found a correlation between 3′ UTR lengthening and differentiation (Shepard et al., 2011).

#### During proliferation and cell transformation

A general shift toward more proximal poly(A) site usage is seen during increased proliferation, leading to 3′ UTR shortening. Enhanced usage of proximal intronic poly(A) sites is also seen, leading to changes in mRNA CRs (Sandberg et al., 2008; Elkon et al., 2012). 3′ UTR shortening has also been reported be associated to cell transformation (Mayr and Bartel, 2009; Singh et al., 2009; Morris et al., 2012), with the transformed cells expressing mRNAs with shorter 3′ UTRs compared with non-transformed cells with similar proliferation rate (Mayr and Bartel, 2009). 3′ UTR shortening has also been associated with poor cancer prognosis (Lembo et al., 2012). 3′ UTR shortening is, e.g., seen for fibroblast growth factor 2 (FGF-2), which is involved in tumor neovascularization. The distal poly(A) site is primarily utilized in fibroblasts, in contrast to in transformed cell lines, where more proximal poly(A) sites are utilized (Touriol et al., 1999). The 3′ UTR shortening is stronger during the transition from arrested to proliferative state than during the transition from proliferative to transformed state (Elkon et al., 2012). Recent studies have found the association between 3′ UTR shortening and cell transformation to be inconsistent. One study found an even distribution between genes with 3′ UTR shortening and lengthening in one transformed cell line (Elkon et al., 2012). Another study found a shift toward usage of more distal poly(A) sites for the majority of genes in another transformed cell line (Fu et al., 2011). Both these studies did however find a general 3′ UTR shortening in other transformed cell lines (Fu et al., 2011; Elkon et al., 2012). Yet other studies found a general 3′ UTR shortening compared to matched normal tissue, both in tumor samples from five different tissues (Lin et al., 2012) and in several colorectal carcinomas (Morris et al., 2012). These examples illustrate that the shifts in poly(A) site usage in transformed cells is more complex than just a shift toward usage of more proximal poly(A) sites.

### The polyadenylation pattern as a biomarker

Poly(A) site usage differs according to tissue type, developmental stage, genotype, and cancer subtype (Breton et al., 2001; Zhang et al., 2005; Kubo et al., 2006; Kwan et al., 2008; Sandberg et al., 2008; Wang et al., 2008; Ji and Tian, 2009; Ji et al., 2009; Singh et al., 2009; Thomsen et al., 2010; Derti et al., 2012; MacIsaac et al., 2012). This makes the polyadenylation pattern a candidate biomarker, which could be used for, e.g., cancer classification. Tissue-specific APA is common and often associated with the proximal non-canonical poly(A) sites (Beaudoing et al., 2000). It has been estimated that 52% of all CR-APA events and 80% of all UTR-APA events are regulated differentially between tissues (Wang et al., 2008), making tissue-specific UTR-APA events even more differentially regulated than tissue-specific alternative splicing.

## Alterations in Polyadenylation in Endocrine Disease

### Sequence changes in and around cis-elements

Changes in the sequence in and around cis-elements can disrupt normal polyadenylation and cause disease (reviewed in Danckwardt et al., 2008). Single nucleotide polymorphisms (SNPs) have been identified that can both create or disturb PASs (Thomas and Sætrom, 2012). SNPs in PASs can affect mRNA length and expression, and are significant predictors of gene expression levels (Thomas and Sætrom, 2012; Yoon et al., 2012). SNPs can also affect APA. A significant high fraction of the SNPs which affect APA, are linked to SNPs found by genome-wide association studies (GWAS). A high proportion of the APA-alleles created by these SNPs, are positively correlated with disease-risk alleles (Thomas and Sætrom, 2012). GWAS-indentified trait-associated SNPs are generally overrepresented in the 3′ UTRs of genes, possibly creating, or affecting the cis-elements involved in polyadenylation (Arnold et al., 2012).

#### Loss of function changes

A functional poly(A) site is required for polyadenylation and thus transcription termination (Connelly and Manley, 1988; West and Proudfoot, 2009). Loss of function changes in the cis-elements of poly(A) sites, can therefore lead to reduced gene expression caused by weakened polyadenylation efficiency. Such loss of function changes are associated with various diseases. They were first described in the PAS of the only poly(A) site in the *HBA2* gene causing α-thalassemias (Higgs et al., 1983; Harteveld et al., 1994) and in a PAS in the *HBB* gene causing β-thalassemias (Orkin et al., 1985; Jankovic et al., 1990; Rund et al., 1992; van Solinge et al., 1996). Loss of function changes causing disease, frequently seem to affect the PAS, which in one of the two core cis-elements found at poly(A) sites. Any possible base change in the canonical PAS (AAUAAA) significantly reduce polyadenylation efficiency (Sheets et al., 1990). The PAS is responsible for binding the core polyadenylation factor CPSF, which is required for polyadenylation (Chan et al., 2011). The reduced efficiency of polyadenylation associated with changes to the canonical PAS, is thus caused by weakened recruitment of CPSF to poly(A) sites. The examples of endocrine diseases affected by loss of function changes in and around cis-elements of poly(A) sites are described below.

##### Neonatal diabetes

A loss of function mutation in the *INS* gene PAS has been shown to cause neonatal diabetes. This disruptive mutation changes the PAS of the only poly(A) site (AAUAAA to AAUAAG), reducing insulin mRNA expression to less than 3 × 10^-4^ percent (Garin et al., 2010). The authors hypothesize that this is due to impaired mRNA stability. However, such a low expression level is more likely a result of weakened polyadenylation efficiency of the only poly(A) site in the *INS* gene, leading to impaired transcription termination and thus reduced gene expression, as seen similarly for other genes (Higgs et al., 1983; Bennett et al., 2001; Stacey et al., 2011). The change of the PAS sequence (AAUAAA to AAUAAG) reduces polyadenylation efficiency about 98% (Sheets et al., 1990). As the PAS is crucial for binding CPSF, the reduced polyadenylation efficiency in the mutated *INS* gene is due to weakened CPSF recruitment (Chan et al., 2011).

##### Immune dysfunction, polyendocrinopathy, enteropathy, X-linked

A loss of function mutation in the PAS of the only poly(A) site in the *FOXP3* gene causes immune dysfunction, polyendocrinopathy, enteropathy, X-linked (IPEX). The mutation changes the PAS (AAUAAA to AAUGAA), leading to diminished FOXP3 mRNA expression (Bennett et al., 2001). This is probably due to impaired polyadenylation of the only poly(A) site in the *FOXP3* gene, leading to non-termination of transcription, as seen similarly for other genes (Higgs et al., 1983; Stacey et al., 2011). The change of the PAS sequence (AAUAAA to AAUGAA) reduces polyadenylation efficiency about 95% (Sheets et al., 1990). The PAS is vital for binding CPSF, hence the impaired polyadenylation of the mutated *FOXP3* gene is due to weakened binding of CPSF (Chan et al., 2011).

##### Mayer–Rokitanski–Küster–Hauser syndrome

The AMH gene encoding Anti-Müllerian Hormone is located only 739 bp downstream of the housekeeping gene encoding splicing factor 3a subunit 2 (SF3a2) (Dresser et al., 1995), a component of the splice factor U2 (Bennett and Reed, 1993). Read-through transcription of the *SF3a2* gene into the *AMH* gene, due to failed polyadenylation of the *SF3a2* gene, is normally seen for a small proportion of transcripts (Dresser et al., 1995). This leads to constitutive expression of AMH along with SF3a2 and results in higher than normal AMH levels. It is hypothesized, that polymorphisms around the *SF3a2* gene poly(A) site leading to reduced polyadenylation with more read-through transcription, could cause Mayer–Rokitanski–Küster–Hauser (MRKH) syndrome. MRKH syndrome is characterized by the Müllerian ducts failing to develop into the uterus, cervix, and upper vagina. Polymorphisms around the *SF3a2* gene poly(A) site were however not found in 30 MRKH patients examined in one study (Oppelt et al., 2005).

##### Hypothalamic-pituitary-adrenal axis dysregulation

In the serotonin transporter (*SERT*) gene a PAS U/G polymorphism exists in the distal, more canonical poly(A) site. The G-allele changes the PAS (AUUAAC to AGUAAC) leading to a lesser usage of the distal poly(A) site. This reduces total SERT expression to about half in the brain (Gyawali et al., 2010). This is sensible as the distal, canonical poly(A) site is most efficient for polyadenylation and most frequently utilized, therefore yielding higher mRNA expression levels (Wang et al., 2013). The G-allele is associated with an increased risk for panic disorder (Gyawali et al., 2010), as well as heightened anxiety and depressive symptoms (Hartley et al., 2012). Interestingly, the SSRI fluoxetine increases the usage of the distal poly(A) site, suggesting that the therapeutic effect of this compound depends on alterations in polyadenylation (Hartley et al., 2012). Serotonin has many functions in the endocrine system, e.g., in the regulation of the hypothalamic-pituitary-adrenal axis (Heisler et al., 2007). This suggests that the *SERT* gene polymorphism could have endocrine implications as well.

##### Cancer

In the PAS of the only poly(A) site in the *TP53* gene a A/C polymorphism exists. The C-allele changes the PAS (AAUAAA to AAUACA), leading to impaired polyadenylation and transcription termination. This results in decreased p53 mRNA expression and read-through trancription. The change of the PAS sequence (AAUAAA to AAUACA) reduces polyadenylation efficiency about 89% (Sheets et al., 1990). CPSF binds the PAS, so the reduced polyadenylation efficiency is due to weakened binding of CPSF (Chan et al., 2011). The C-allele is associated with various cancer diseases (Stacey et al., 2011; Zhou et al., 2012). This polymorphism could be important for endocrine tumor diseases as well, as loss of p53 is an important event in the pathogenesis of many tumors, e.g., thyroid tumors (Yoshimoto et al., 1992).

#### Gain of function changes

Gain of function changes in the cis-elements of poly(A) sites, can lead to enhanced gene expression caused by improved polyadenylation efficiency and to changes in APA. Such gain of function changes are associated with various diseases. They were first described in the CA dinucleotide immediately 5′ of the cleavage site in the *F2* gene encoding thrombin (Gehring et al., 2001; Ceelie et al., 2004; Danckwardt et al., 2004, 2006, 2007). Later they were also described in the DSE of the *F2* gene (Danckwardt et al., 2004), as well as in the DSE of the *FGG* gene encoding fibrinogen gamma (Lisman et al., 2005; Uitte de Willige et al., 2007). All of these mutations cause thrombophilia through enhanced polyadenylation efficiency, leading to upregulated gene expression. The examples of endocrine diseases affected by gain of function changes in and around cis-elements of poly(A) sites are described below.

##### Type I diabetes

The *GIMAP5* gene has two poly(A) sites in its 3′ UTR. In the PAS of the proximal poly(A) site a G/A polymorphism exists. The minor A-allele changes the PAS to a canonical one (AAUAGA to AAUAAA) leading to enhanced proximal poly(a) site usage (Shin et al., 2007). The change of the PAS sequence (AAUAGA to AAUAAA) increases polyadenylation efficiency about 25-fold (Sheets et al., 1990). The A-allele is associated with high levels of tyrosine phosphatase (IA-2) autoantibodies, which are reported to be associated with clinical onset of type I diabetes (Shin et al., 2007). Interestingly, the G-allele is associated with an increased risk of systemic lupus erythematosus (SLE) (Hellquist et al., 2007). The A-allele induce a higher proportion of mRNAs with the short 3′ UTR isoform, but was not found to have an effect on total GIMAP5 mRNA levels (Hellquist et al., 2007). Differences in translational efficiency between the mRNAs with the long and short 3′ UTR isoforms are thus believed to be affecting the levels of IA-2 autoantibodies.

##### Type II diabetes

A proximal intronic poly(A) site in the transcription factor 7-like 2 (*TCF7L2*) gene has been found. Usage of this poly(A) site leads to production of a C-terminally truncated protein isoform that repress T-cell factor/lymphoid-enhancer factor (TCF/LEF)-dependent target genes (Locke et al., 2011). The authors hypothesize that this poly(A) site might explain why intronic SNPs in the *TCF7L2* gene are associated with a risk of type 2 diabetes, as they could enhance the utilization of this poly(A) site.

##### IGF-1 deficiency

A U/A polymorphism in a proximal poly(A) site PAS of the insulin-like growth factor 1 (*IGF-1*) gene 3′ UTR has been described. It changes the PAS (AAUAUA to AAAAUA), leading to expression of a shorter than normal IGF-1 mRNA. The U/A polymorphism has been reported in a child born small for gestation age with IGF-1 deficiency (Bonapace et al., 2003). A later study however reported that this polymorphism along with other polymorphisms in this PAS of the *IGF-1* gene do not cause IGF-1 deficiency nor growth impairment (Coutinho et al., 2007).

##### Endocrine tumor diseases

A C/T polymorphism in the *RET* proto-oncogene, six nucleotides upstream of an intronic proximal poly(A) site PAS of intron 19 exists (Gartner et al., 2005). The adjacent poly(A) site is commonly used for polyadenylation by CR-APA. The C/T polymorphism lies within a binding site of the nucleic acid binding protein Pbx1 (AAATTAG(C/T)T), which might affect polyadenylation at the adjacent poly (A) site. The T-allele carriers have the more canonical binding motif for Pbx1. Heterozygosity for the T-allele was found in a significantly higher number of patients with various endocrine tumors than in healthy controls. Homozygosity for the T-allele was exclusively found in DNA from endocrine tumors with a high malignant potential, as a result of loss of heterozygosity (LOH) in these tumors (Gartner et al., 2005). This suggests that Pbx1 enhances polyadenylation at the adjacent poly(A) site, leading to higher expression levels of RET.

##### Cancer

Gain of function mutations have been found in the *CCND1* gene encoding Cyclin D1 in mantle cell lymphoma (MCL) tumors. These mutations create new proximal poly(A) sites, giving rise to expression of truncated mRNAs lacking most of the 3′ UTR. These truncated mRNAs are associated with strongly proliferative MCL tumors and inferior survival (Wiestner et al., 2007). Such mutations could be important for endocrine tumor diseases as well, as high expression of Cyclin D1 is associated with many cancers, e.g., pancreatic endocrine tumors (Guo et al., 2003).

### Sequence changes in genes encoding polyadenylation factors

#### Cancer

Mutations in yeast polydadenylation factors have been shown to disrupt normal transcription termination (Birse et al., 1998) and to cause inefficient transcription elongation (Luna et al., 2005). They have also been shown to cause genome instability, through formation of R-loops (Stirling et al., 2012). R-loops are persistent, transcription-associated RNA:DNA hybrids that expose damage-prone single stranded DNA (ssDNA) on the nonsense strand and may block the replication fork progression (Stirling et al., 2012). Non-terminated pre-mRNA transcripts, which form due to defective cleavage at the poly(A) site (Birse et al., 1998), could form the basis of these RNA:DNA hybrids found in R-loops. Genome instability is found in many human cancers, in part due to increased chromosome instability (CIN), which is linked to R-loops (Wahba et al., 2011). CIN is normally found as an early event in the oncogenic process and can lead to loss or gain of whole chromosomes or chromosomal fragments. Researchers working with yeast strains, each with mutations in one gene out of 305 genes involved with CIN, found that mutations in 44 of the genes resulted in an indicated increased CIN. Seven of these 44 genes encoded subunits of polyadenylation factors. They were homologs of CPSF-subunits CPSF-100, hFip1, and Wdr33, CstF subunit CstF-64, both subunits of CF-II and yeast factor HRP1, lacking a human homolog. The mutant stains had indicated increased CIN in all cell cycle stages (Stirling et al., 2012). Genome-wide analysis of fragile chromatin sites in these mutant stains supported a transcription-dependent mechanism of DNA damage, characteristic of R-loop formation. Increased RNA:DNA hybrid formation in polyadenylation factor mutant stains were found and the resulting CIN could be suppressed by RNaseH, which degrades the RNA and thereby removes the R-loops (Stirling et al., 2012). The researchers found that small interfering RNA (siRNA) knockdown of the CPSF-subunit hFip1 in human cells increased DNA damage and chromosome breakage (Stirling et al., 2012). Interestingly, hFip1 also comprises the N-terminal part of a fusion protein with platelet-derived growth factor receptor alpha (PDGFRα), which causes 14–60% the incidents of hypereosinophilic syndrome/chronic eosinophilic leukemia (Cools et al., 2003; Gotlib and Cools, 2008; Li et al., 2012a). Truncation fusions of yeast FIP1, analogous to those found for hFip1 in chronic eosinophilic leukemia, was found to cause genome instability in yeast (Stirling et al., 2012). All these findings indicate that the normal 3′-end processing complex maintains genome integrity by suppressing R-loop formation, thereby suppressing CIN, a function that may be impaired in many human cancers (Stirling et al., 2012). Mutations in genes encoding polyadenylation factors could be important for endocrine tumor diseases, as CIN is already known as a powerful prognostic indicator for endocrine pancreatic tumor patients (Jonkers et al., 2007).

### Sequence changes in genes encoding regulatory factors

#### Hereditary and sporadic parathyroid tumors

The tumor suppressor protein Cdc73 is mutationally inactivated in hereditary and sporadic parathyroid tumors (Rozenblatt-Rosen et al., 2009). It normally associates with CPSF-CstF complexes and enhances polyadenylation of specific target genes. The inactivation of Cdc73 leads to decreased association of CPSF-CstF complexes with these target genes, reducing their expression levels (Rozenblatt-Rosen et al., 2009). This suggests that impaired polyadenylation of these target genes plays a part in the parathyroid tumorigenesis.

### Other mechanisms leading to alterations in polyadenylation

#### Fragile X-associated premature ovarian insufficiency

A shift in poly(A) site usage due to UTR-APA is seen in the fragile X mental retardation 1 (*FMR1*) gene premutation alleles compared to normal alleles (Tassone et al., 2011). The *FMR1* gene 5′ UTR contains a CGG repeat element that is expanded to more than 200 CGG repeats and methylated in fragile X syndrome. The premutation alleles of the *FMR1* gene, with 55–200 CGG repeats, are associated with various phenotypes, including fragile X-associated premature ovarian insufficiency (Toniolo and Rizzolio, 2007). These phenotypic manifestations could be a result of the UTR-APA of the FMR1 pre-mRNA. The choice of alternative 5′ UTRs affects the choice of poly(A) site (Winter et al., 2007; Ji et al., 2011). This could explain how the CGG expansion in the 5′ UTR of the *FMR1* gene affects the choice of poly(A) site by altering the transcription start site (Tassone et al., 2011).

#### Diabetic nephropathy

A high-glucose-regulated gene, *HGRG-14*, found in human mesangial cells, is expressing a longer isoform of HGRG-14 mRNA due to UTR-APA under high-glucose conditions (Abdel Wahab et al., 1998). This results in lower HGRG-14 protein levels. The switch from the shorter to the longer isoform is detected within 2 h of exposure to high-glucose levels and the authors hypothesize that this may be involved in the pathogenesis of diabetic nephropathy (Abdel Wahab et al., 1998).

#### Medullary thyroid carcinoma

Human Thyroid C cells express an alternative isoform of calcitonin mRNA, containing all exons of the *CALCA* gene, producing calcitonin, and calcitonin carboxyl terminal peptide II (CCP II). Expression of this isoform is seen in low levels in normal subjects. In medullary thyroid carcinomas expression is more abundant, leading to higher plasma levels of CCP II (Minvielle et al., 1991). CCP II could thus be used as a diagnostic marker for medullary thyroid carcinomas. As the alternative processing of the *CALCA* gene occurs through APA, polyadenylation regulation may be aberrant in the medullary thyroid carcinomas.

#### Pre-eclampsia

Soluble fms-like tyrosine kinase 1 (sFlt-1) arises from CR-APA utilizing an intronic poly(A) site in the *FLT1* gene encoding VEGF receptor 1 (Thomas et al., 2007). sFlt-1 binds VEGF and thereby reduces its free circulating level. Expression of sFlt-1 is upregulated in cytotrophoblasts during hypoxia and is suggested to induce endothelial damage (Nagamatsu et al., 2004; Zhao et al., 2012). sFlt-1 expression is increased in pre-eclampsia and evidence suggests that it causes the development of the disease (Shibata et al., 2005; Zhao et al., 2012). Selective expression of sFlt-1 can be induced by targeting the 5′ splice site with an antisense oligonucleotide, thereby inhibiting U1 binding (Vorlová et al., 2011). This hints that U1 plays a role in the upregulation of sFlt-1. Several techniques using antisense elements can inhibit poly(A) site usage (see section *Treatment by altering polyadenylation*) (Beckley et al., 2001; Fortes et al., 2003; Goraczniak et al., 2009; Vickers et al., 2011; Vorlová et al., 2011; Blazquez et al., 2012; Vickers and Crooke, 2012). These techniques could be used to lower the expression of sFlt-1, possibly alleviating the symptoms or even treating pre-eclampsia in the future. Interestingly, sFlt-1 is also increased in type II diabetes (Nandy et al., 2010).

#### Ectopic Cushing syndrome

Ectopic Cushing syndrome is cause by adrenocorticotropic hormone (ACTH) production in non-pituitary tumors, such as small cell lung cancers, which is normally non-suppressible (Parks et al., 1998). An ACTH-producing small cell lung cancer was shown to express a C-terminally truncated isoform of glucocorticoid receptor due to CR-APA using an intronic poly(A) site. This caused the ACTH production to be non-suppressible by exogenous glucocorticoid administration (Parks et al., 1998). Several techniques using antisense elements can inhibit poly(A) site usage (see Treatment by altering polyadenylation) (Beckley et al., 2001; Fortes et al., 2003; Goraczniak et al., 2009; Vickers et al., 2011; Vorlová et al., 2011; Blazquez et al., 2012; Vickers and Crooke, 2012). If these techniques were used to target the intronic poly(A) site utilized by CR-APA, the normal full length isoform of glucocorticoid receptor would be expressed. This would render the ACTH production suppressible by glucocorticoids, possibly treating the Cushing syndrome.

#### Adrenal and gonadal dysfunction

The gene encoding the steroidogenic acute regulatory (StAR) protein express two mRNA isoforms (1.6 and 3.5 kb) differing only in the length of their 3′ UTR due to UTR-APA (Duan et al., 2009). StAR regulates the rate-limiting step in steroid biosynthesis. ACTH stimulates the StAR function by increasing cAMP and cAMP-protein kinase A (PKA) activity, leading to preferential expression of the longer, less stable 3.5 kb StAR mRNA isoform (Zhao et al., 2005). PKA also stimulate transcription of the RBP TIS11b, which selectively binds the 3.5 kb mRNA isoform and destabilizes it, while at the same time enhancing StAR protein translation (Duan et al., 2009). Aberrant expression of StAR due to alterations in polydenylation could cause abnormal steroid production and adrenal/gonadal dysfunction, as seen for other disruptive mutations leading to enhanced or diminished StAR expression (Okuhara et al., 2008; Sahakitrungruang et al., 2010).

## Treatment by Altering Polyadenylation

### Antisense elements targeting specific poly(A) sites

#### Enhancing poly(A) site usage

Specific usage of selected intronic poly(A) sites can be induced by targeting the upstream 5′ splice site with an antisense oligonucleotide, thereby inhibiting U1 binding (Vorlová et al., 2011). As U1 associating with 5′ splice sites represses adjacent downstream intronic poly(A) site, this enhances the usage of these intronic poly(A) sites and leads to expression of C-terminal truncated proteins (Kaida et al., 2010; Vorlová et al., 2011; Berg et al., 2012). Unlike mRNAs with acquired premature termination codons, which also generate C-terminal truncated proteins, the mRNAs generated by usage of intronic poly(A) sites are not targeted by nonsense-mediated decay (Lejeune and Maquat, 2005). Thousands of dormant intronic poly(A) sites have been characterized (Yao et al., 2012a). Induced usage of any of these sites can lead to the expression of specific C-terminal truncated protein isoforms.

#### Inhibiting poly(A) site usage

Different techniques using antisense elements have been used to effectively inhibit poly(A) site usage. They include antisense oligonucleotides (Vorlová et al., 2011), siRNAs (Vickers and Crooke, 2012), and U1 modifications (Beckley et al., 2001; Fortes et al., 2003; Goraczniak et al., 2009; Vickers et al., 2011; Blazquez et al., 2012). The techniques can both induce APA and lead to significantly reduced gene expression, depending on the poly(A) site targeted.

### Drugs targeting polyadenylation factors

The specific PAP inhibitor cordycepin is an adenosine-nucleotide analog. It reduces polyadenylation efficiency and causes defects in transcription termination (Kondrashov et al., 2012). Cordycepin has been shown to inhibit proliferation and induce apoptosis in various cancer cell lines (Thomadaki et al., 2008; Imesch et al., 2011). Cordycepin also inhibit the induction of inflammatory genes mediated by cytokines (Kondrashov et al., 2012).

## Perspectives

Recently, the field of polyadenylation has seen major progress. Advances in high-throughput sequencing have made it possible to characterize polyadenylation genome-wide and lowered the price to ∼100$ per sample (Wang et al., 2013). This has stimulated the field and more research on polyadenylation is now being published. This will help to find more endocrine pathways regulated by polyadenylation and thus to discover novel implications of alterations in polyadenylation for endocrine disease. The lowered cost of genome-wide characterization of polyadenylation will also push forward the implementation of this technique into the clinic to improve diagnostics. For instance, cancers show characteristic changes in APA patterns (Mayr and Bartel, 2009; Singh et al., 2009; Fu et al., 2011; Elkon et al., 2012; Lin et al., 2012; Morris et al., 2012). The inclusion of APA screenings in diagnostics could therefore help to produce more accurate diagnoses by, e.g., differentiating between cancer subtypes. It is therefore crucial to increase the awareness about the importance of polyadenylation among physicians and scientists working with endocrinology.

Alternative polyadenylation is increasingly being recognized as an important regulator of the human transcriptome, like alternative splicing. It has been estimated that 52% of all CR-APA events and 80% of all UTR-APA events are regulated differentially between tissues, making tissue-specific UTR-APA events even more differentially regulated than tissue-specific alternative splicing (Wang et al., 2008). Interestingly, changes in the APA pattern is seen during changes in cellular conditions, such as proliferation, differentiation, cellular transformation, and dedifferentiation (Sandberg et al., 2008; Ji and Tian, 2009; Mayr and Bartel, 2009; Singh et al., 2009; Fu et al., 2011; Elkon et al., 2012; Lin et al., 2012; Morris et al., 2012). Unlike mRNAs created by alternative splicing, mRNAs generated by CR-APA are not targeted by nonsense-mediated decay, thus lead to the production of C-terminally truncated protein isoforms (Lejeune and Maquat, 2005). Such isoforms can have significantly altered functions (Vorlová et al., 2011) and have been suggested to cause diseases such as pre-ecclamsia (Zhao et al., 2012). C-terminally truncated protein isoforms generated by CR-APA could be causative of many other diseases. UTR-APA alters the 3′ UTR length and induces changes in stability and translational efficiency for individual mRNAs (Sandberg et al., 2008; Mayr and Bartel, 2009; Singh et al., 2009; Hogg and Goff, 2010; Yepiskoposyan et al., 2011). However, the effect of global changes in 3′ UTR length remains mysterious as no significant change in mRNA levels were seen in several cases with general changes in 3′ UTR length (Sandberg et al., 2008; Fu et al., 2011; Elkon et al., 2012; Morris et al., 2012). The latter findings could be explained by the mRNAs competing for trans-acting factors. For instance, in activated neurons, a general upregulation of pre-mRNAs, leads to a relative shortage of splice factor U1, which in turn, leads to changes in APA (Berg et al., 2012). Similarly, a general change in mRNA 3′ UTR length could lead to a relative shortage/abundance of trans-acting factors, giving rise to a relative weaker effect on gene expression levels, than seen for a single gene expressing an alternative 3′ UTR isoform. It would however still be interesting to investigate the changes in protein translation efficiency in cells with general changes in 3′ UTR length.

Both loss of function and gain of function changes in and around poly(A) sites have been shown to cause disease (reviewed in Danckwardt et al., 2008). It is probable that loss of function changes most frequently cause disease, when affecting the stronger, more canonical poly(A) site of a gene. Firstly, these sites are most efficient for polyadenylation and the most commonly utilized. Usage of these sites therefore give rise to higher mRNA expression levels (Wang et al., 2013). Secondly, these sites are often found as the most distal poly(A) site, which works as the last option for polyadenylation and ensures that transcription is terminated (Beaudoing et al., 2000; Tian et al., 2005, 2007; Shepard et al., 2011; de Klerk et al., 2012; Lin et al., 2012; Yoon et al., 2012; Wang et al., 2013). When loss of function changes affect these more canonical poly(A) sites, the effect on gene expression is therefore strong. This is seen for the *SERT* gene encoding a SERT. Here a loss of function change in the PAS of the distal, canonical poly(A) site, changes the PAS (AUUAAC to AGUAAC). This leads to lesser usage of the poly(A) site, reducing total SERT expression to about half (Gyawali et al., 2010). When loss of function changes affect the poly(A) site in genes where only one poly(A) site is found, the effect on gene expression is even stronger, as no alternative poly (A) sites may be used. This is seen for the *INS*, *FOXP3*, and *TP53* genes, where loss of function changes in the PAS of the only poly(A) site in these genes, lead to an almost diminished gene expression (Bennett et al., 2001; Garin et al., 2010; Stacey et al., 2011). When loss of function changes affect the weaker poly(A) sites in genes with multiple poly(A) sites, the effect is changes in the APA pattern, altering the 3′ UTR and in some cases the protein product. Gene expression is also affected, but often in a more subtle way. Interestingly, loss of function changes causing disease also commonly seem to affect the PAS (Higgs et al., 1983; Bennett et al., 2001; Garin et al., 2010; Gyawali et al., 2010; Stacey et al., 2011). This is predictable, as the PAS is one of the two core cis-elements found at poly(A) sites. It is essential for binding CPSF, which is required for both the cleavage and the polyadenylation step (Chan et al., 2011). It is important to note that loss of function changes can also lead to subclinical manifestations, as seen in the metachromatic leukodystrophy pseudodeficiency phenotype. Here a change in the PAS (AAUAAC to AGUAAC) of a majorly used proximal poly(A) site in the *ARSA* gene reduces gene expression by 90%(Gieselmann et al., 1989). Conversely, it seems that gain of function changes associated with disease often affect proximal poly(A) sites. This is expected, as the proximal poly(A) sites are generally weaker and therefore have the biggest potential to be enhanced (Beaudoing et al., 2000; Tian et al., 2005, 2007; Shepard et al., 2011; de Klerk et al., 2012; Lin et al., 2012; Yoon et al., 2012; Wang et al., 2013). Interestingly, gain of function changes causing disease also commonly seem to affect other cis-elements than the PAS, as seen for the CA dinucleotide immediately 5′ of the cleavage site and for the DSE (Gehring et al., 2001; Ceelie et al., 2004; Danckwardt et al., 2004, 2006, 2007; Uitte de Willige et al., 2007). This is also anticipated, as improvement of auxiliary cis-elements can both enhance polyadenylation at poly(A) sites with a canonical PAS and at poly(A) sites with a non-canonical PAS.

Various techniques using specific antisense elements that enhance or inhibit the usage of specific poly(A) sites exists (Beckley et al., 2001; Fortes et al., 2003; Goraczniak et al., 2009; Vickers et al., 2011; Vorlová et al., 2011; Blazquez et al., 2012; Vickers and Crooke, 2012). These techniques hold great promises of novel therapeutic approaches, as they can both induce APA and change gene expression significantly. One obvious use would be to reverse the effect of disease-causing mutations affecting polyadenylation. However, they could also be used to treat diseases, where alterations in polyadenylation do not take part in the pathogenesis. One way of treating could be by inducing the expression of C-terminally truncated protein isoforms. This primarily leads to loss of function of the full length product, but the expressed C-terminally truncated protein isoforms can have beneficial functions on their own. This is seen for the VEGF receptor, where a C-terminally truncated isoform functions as a soluble decoy. Induction of this isoform resulted in an antiangiogenic effect both in targeted cells and in untreated cells exposed to the conditioned media (Vorlová et al., 2011). Another way to treat using these techniques, is by significantly reducing gene expression by inhibiting the more canonical poly(A) site (Goraczniak et al., 2009; Blazquez et al., 2012). A potential side-effect free usage of these techniques, would be to inhibit premature intronic poly(A) sites, which are used in diseased cells, but not in healthy cells. As polyadenylation is inhibited at the intronic poly(A) site, the targeted intron will be spliced out and degraded, leaving the healthy cells unaffected by the treatment.

In conclusion, the clinical implications of the current state of research in this field are: (1) Poly(A) site usage differs according to tissue type, developmental stage, genotype, and cancer subtype (Breton et al., 2001; Zhang et al., 2005; Kubo et al., 2006; Kwan et al., 2008; Sandberg et al., 2008; Wang et al., 2008; Ji and Tian, 2009; Ji et al., 2009; Singh et al., 2009; Thomsen et al., 2010; Derti et al., 2012; MacIsaac et al., 2012). Characterization of polyadenylation can therefore improve diagnostics and poly(A) site usage would be a candidate diagnostic biomarker for, e.g., cancer classification. (2) Many diseases and symptoms are caused by defective polyadenylation (reviewed in Danckwardt et al., 2008). Several endocrine diseases are either caused by or associated with alterations in polyadenylation. These are neonatal diabetes (Garin et al., 2010), IPEX (Bennett et al., 2001), type I and II diabetes (Shin et al., 2007; Locke et al., 2011), pre-eclampsia (Zhao et al., 2012), fragile X-associated premature ovarian insufficiency (Tassone et al., 2011), ectopic Cushing syndrome (Parks et al., 1998), and several types of endocrine tumor diseases (Minvielle et al., 1991; Gartner et al., 2005). Consequently, the advances in this field therefore lead to a better understanding of these diseases and expose new possible drug targets. (3) Novel techniques using antisense elements that can both enhance and inhibit the usage of specific poly(A) sites have been developed (Beckley et al., 2001; Fortes et al., 2003; Goraczniak et al., 2009; Vickers et al., 2011; Vorlová et al., 2011; Blazquez et al., 2012; Vickers and Crooke, 2012). These techniques hold the promise of novel therapeutic approaches, through desired induced changes in polyadenylation.

## Authors Contribution

Drafting of the manuscript (Anders Rehfeld, Lennart Friis-Hansen), critical revision of the manuscript (Anders Rehfeld, Mireya Plass, Anders Krogh, Lennart Friis-Hansen), and obtained funding (Anders Rehfeld, Anders Krogh, Lennart Friis-Hansen).

## Conflict of Interest Statement

The authors declare that the research was conducted in the absence of any commercial or financial relationships that could be construed as a potential conflict of interest.

## Abbreviations

3′ UTR, 3′ untranslated region; APA, alternative polyadenylation; ARE, AU-rich element; Aux-DSE, auxiliary downstream element; CBC, nuclear cap-binding complex; CF-I and CF-II, cleavage factor I and II; CGRP, calcitonin gene-related peptide; CIN, chromosome instability; CPSF, cleavage and polyadenylation specificity factor; CR-APA, coding region-APA; CstF, cleavage stimulation factor; CTD, C-terminal domain; DSE, downstream sequence element; FMR1, fragile X mental retardation 1; GRE, GU-rich element; GWAS, genome-wide association studies; HSF1, heat shock factor 1; IA-2, tyrosine phosphatase; IGF-1, insulin-like growth factor 1; IPEX, immune dysfunction, polyendocrinopathy, enteropathy, X-linked; LOH, loss of heterozygosity; MCL, mantle cell lymphoma; miRNA, micro-RNA; mRNA, messenger RNA; PABPN1, nuclear poly(A)-binding protein; PAP, poly(A) polymerase; PARP1, poly(ADP-ribose) polymerase 1; PAS, polyadenylation signal; PDGFRα, platelet-derived growth factor receptor alpha; PI4,5P2, phosphatidylinositol 4,5-bisphosphate; Pol-II, RNA-polymerase II; PTB, polypyrimidine tract binding protein; RBP, RNA-binding protein; RET, rearranged during transfection; Ser2, Serine 2; SERT, serotonin transporter; SF3a2, splicing factor 3a subunit 2; sFlt-1, soluble fms-like tyrosine kinase 1; siRNA, small interfering RNA; SLE, systemic lupus erythematosus; SNP, single nucleotide polymorphisms; SOCS3, suppressor of cytokine signaling 3; TB-RBP, testis brain RNA-binding protein; TCF/LEF, T-cell factor/lymphoid-enhancer factor; TCF7L2, transcription factor 7-like 2; U2AF1 and U2AF2, U2 auxiliary factor 1 and 2; USE, upstream sequence element; UTR-APA, untranslated region-APA.

## References

[B1] Abdel Wahab N. Gibbs J. Mason R. M. (1998). Regulation of gene expression by alternative polyadenylation and mRNA instability in hyperglycaemic mesangial cells. Biochem. J. 336(Pt 2), 405–411 9820818 10.1042/bj3360405 PMC1219885

[B2] Ahn S. H. Kim M. Buratowski S. (2004). Phosphorylation of serine 2 within the RNA polymerase II C-terminal domain couples transcription and 3′ end processing. Mol. Cell 13, 67–76 10.1016/S1097-2765(03)00492-1 14731395

[B3] Akhtar M. N. Bukhari S. A. Fazal Z. Qamar R. Shahmuradov I. A. (2010). POLYAR, a new computer program for prediction of poly(A) sites in human sequences. BMC Genomics 11:646 10.1186/1471-2164-11-646 21092114 PMC3053588

[B4] Akman B. H. Can T. Erson-Bensan A. E. (2012). Estrogen-induced upregulation and 3′-UTR shortening of CDC6. Nucleic Acids Res. 40, 10679–10688 10.1093/nar/gks855 22977174 PMC3510512

[B5] Amara S. G. Evans R. M. Rosenfeld M. G. (1984). Calcitonin/calcitonin gene-related peptide transcription unit: tissue-specific expression involves selective use of alternative polyadenylation sites. Mol. Cell. Biol. 4, 2151–2160 6334229 10.1128/mcb.4.10.2151-2160.1984 PMC369034

[B6] Ara T. Lopez F. Ritchie W. Benech P. Gautheret D. (2006). Conservation of alternative polyadenylation patterns in mammalian genes. BMC Genomics 7:189 10.1186/1471-2164-7-189 16872498 PMC1550727

[B7] Arnold M. Ellwanger D. C. Hartsperger M. L. Pfeufer A. Stümpflen V. (2012). Cis-acting polymorphisms affect complex traits through modifications of microRNA regulation pathways. PLoS ONE 7:e36694 10.1371/journal.pone.0036694 22606281 PMC3350471

[B8] Bajic V. B. Charn T. H. Xu J. X. Panda S. K. Krishnan T. S. P. (2005). Prediction models for DNA transcription termination based on SOM networks. Conference proceedings: Annual International Conference of the IEEE Engineering in Medicine and Biology Society. IEEE Engineering in Medicine and Biology Society. Conference, Shanghai, 5, 4791–4 10.1109/IEMBS.2005.1615543 17281313

[B9] Barabino S. M. Keller W. (1999). Last but not least: regulated poly(A) tail formation. Cell 99, 9–11 10.1016/S0092-8674(00)80057-4 10520989

[B10] Beaudoing E. Freier S. Wyatt J. R. Claverie J. M. Gautheret D. (2000). Patterns of variant polyadenylation signal usage in human genes. Genome Res. 10, 1001–1010 10.1101/gr.10.7.1001 10899149 PMC310884

[B11] Beckley S. A. Liu P. Stover M. L. Gunderson S. I. Lichtler A. C. Rowe D. W. (2001). Reduction of target gene expression by a modified U1 snRNA. Mol. Cell. Biol. 21, 2815–2825 10.1128/MCB.21.8.2815-2825.2001 11283260 PMC86911

[B12] Bennett C. L. Brunkow M. E. Ramsdell F. O’Briant K. C. Zhu Q. Fuleihan R. L. (2001). A rare polyadenylation signal mutation of the FOXP3 gene (AAUAAA → AAUGAA) leads to the IPEX syndrome. Immunogenetics 53, 435–439 10.1007/s002510100358 11685453

[B13] Bennett M. Reed R. (1993). Correspondence between a mammalian spliceosome component and an essential yeast splicing factor. Science 262, 105–108 10.1126/science.8211113 8211113

[B14] Berg M. G. Singh L. N. Younis I. Liu Q. Pinto A. M. Kaida D. (2012). U1 snRNP determines mRNA length and regulates isoform expression. Cell 150, 53–64 10.1016/j.cell.2012.05.029 22770214 PMC3412174

[B15] Bhattacharjee R. B. Bag J. (2012). Depletion of nuclear poly(A) binding protein PABPN1 produces a compensatory response by cytoplasmic PABP4 and PABP5 in cultured human cells. PLoS ONE 7:e53036 10.1371/journal.pone.0053036 23300856 PMC3534090

[B16] Birse C. E. Minvielle-Sebastia L. Lee B. A. Keller W. Proudfoot N. J. (1998). Coupling termination of transcription to messenger RNA maturation in yeast. Science 280, 298–301 10.1126/science.280.5361.298 9535662

[B17] Blazquez L. Gonzalez-Rojas S. J. Abad A. Razquin N. Abad X. Fortes P. (2012). Increased in vivo inhibition of gene expression by combining RNA interference and U1 inhibition. Nucleic Acids Res. 40, e8 10.1093/nar/gkr956 22086952 PMC3245954

[B18] Blechingberg J. Lykke-Andersen S. Jensen T. H. Jørgensen A. L. Nielsen A. L. (2007). Regulatory mechanisms for 3′-end alternative splicing and polyadenylation of the Glial Fibrillary Acidic Protein, GFAP, transcript. Nucleic Acids Res. 35, 7636–7650 10.1093/nar/gkm931 17981838 PMC2190720

[B19] Bonapace G. Concolino D. Formicola S. Strisciuglio P. (2003). A novel mutation in a patient with insulin-like growth factor 1 (IGF1) deficiency. J. Med. Genet. 40, 913–917 10.1136/jmg.40.12.913 14684690 PMC1735341

[B20] Boutaud O. Dixon D. A. Oates J. A. Sawaoka H. (2003). Tristetraprolin binds to the COX-2 mRNA 3′ untranslated region in cancer cells. Adv. Exp. Med. Biol. 525, 157–160 10.1007/978-1-4419-9194-2_32 12751757

[B21] Boutet S. C. Cheung T. H. Quach N. L. Liu L. Prescott S. L. Edalati A. (2012). Alternative polyadenylation mediates microRNA regulation of muscle stem cell function. Cell Stem Cell 10, 327–336 10.1016/j.stem.2012.01.017 22385659 PMC3306803

[B22] Breton C. Di Scala-Guenot D. Zingg H. H. (2001). Oxytocin receptor gene expression in rat mammary gland: structural characterization and regulation. J. Mol. Endocrinol. 27, 175–189 10.1677/jme.0.0270175 11564602

[B23] Brown K. M. Gilmartin G. M. (2003). A mechanism for the regulation of pre-mRNA 3′ processing by human cleavage factor Im. Mol. Cell 12, 1467–1476 10.1016/S1097-2765(03)00453-2 14690600

[B24] Buchert M. Papin M. Bonnans C. Darido C. Raye W. S. Garambois V. (2010). Symplekin promotes tumorigenicity by up-regulating claudin-2 expression. Proc. Natl. Acad. Sci. U.S.A. 107, 2628–2633 10.1073/pnas.0903747107 20133805 PMC2823888

[B25] Castelo-Branco P. Furger A. Wollerton M. Smith C. Moreira A. Proudfoot N. (2004). Polypyrimidine tract binding protein modulates efficiency of polyadenylation. Mol. Cell. Biol. 24, 4174–4183 10.1128/MCB.24.10.4174-4183.2004 15121839 PMC400487

[B26] Ceelie H. Spaargaren-van Riel C. C. Bertina R. M. Vos H. L. (2004). G20210A is a functional mutation in the prothrombin gene; effect on protein levels and 3′-end formation. J. Thromb. Haemost. 2, 119–127 10.1111/j.1538-7836.2003.00493.x 14717975

[B27] Chan S. Choi E.-A. Shi Y. (2011). Pre-mRNA 3′-end processing complex assembly and function. Wiley Interdiscip. Rev. RNA 2, 321–335 10.1002/wrna.54 21957020 PMC3980678

[B28] Chen F. Wilusz J. (1998). Auxiliary downstream elements are required for efficient polyadenylation of mammalian pre-mRNAs. Nucleic Acids Res. 26, 2891–2898 10.1093/nar/26.23.v 9611233 PMC147640

[B29] Chen J.-M. Férec C. Cooper D. N. (2006a). A systematic analysis of disease-associated variants in the 3′ regulatory regions of human protein-coding genes I: general principles and overview. Hum. Genet. 120, 1–21 10.1007/s00439-006-0180-7 16645853

[B30] Chen J.-M. Férec C. Cooper D. N. (2006b). A systematic analysis of disease-associated variants in the 3′ regulatory regions of human protein-coding genes II: the importance of mRNA secondary structure in assessing the functionality of 3′ UTR variants. Hum. Genet. 120, 301–333 10.1007/s00439-006-0180-7 16807757

[B31] Cheng Y. Miura R. M. Tian B. (2006). Prediction of mRNA polyadenylation sites by support vector machine. Bioinformatics 22, 2320–2325 10.1093/bioinformatics/btl102 16870936

[B32] Chennathukuzhi V. M. Lefrancois S. Morales C. R. Syed V. Hecht N. B. (2001). Elevated levels of the polyadenylation factor CstF 64 enhance formation of the 1kB Testis brain RNA-binding protein (TB-RBP) mRNA in male germ cells. Mol. Reprod. Dev. 58, 460–469 10.1002/1098-2795(20010401)58:4<460::AID-MRD15>3.0.CO;2-F 11241784

[B33] Connelly S. Manley J. L. (1988). A functional mRNA polyadenylation signal is required for transcription termination by RNA polymerase II. Genes Dev. 2, 440–452 10.1101/gad.2.4.440 2836265

[B34] Cooke C. Alwine J. C. (1996). The cap and the 3′ splice site similarly affect polyadenylation efficiency. Mol. Cell. Biol. 16, 2579–2584 8649365 10.1128/mcb.16.6.2579 PMC231248

[B35] Cools J. DeAngelo D. J. Gotlib J. Stover E. H. Legare R. D. Cortes J. (2003). A tyrosine kinase created by fusion of the PDGFRA and FIP1L1 genes as a therapeutic target of imatinib in idiopathic hypereosinophilic syndrome. N. Engl. J. Med. 348, 1201–1214 10.1056/NEJMoa025217 12660384

[B36] Coutinho D. C. Coletta R. R. D. Costa E. M. F. Pachi P. R. Boguszewski M. C. S. Damiani D. (2007). Polymorphisms identified in the upstream core polyadenylation signal of IGF1 gene exon 6 do not cause pre- and postnatal growth impairment. J. Clin. Endocrinol. Metab. 92, 4889–4892 10.1210/jc.2007-1661 17895313

[B37] Dalziel M. Nunes N. M. Furger A. (2007). Two G-rich regulatory elements located adjacent to and 440 nucleotides downstream of the core poly(A) site of the intronless melanocortin receptor 1 gene are critical for efficient 3′ end processing. Mol. Cell. Biol. 27, 1568–1580 10.1128/MCB.01821-06 17189425 PMC1820467

[B38] Danckwardt S. Gantzert A.-S. Macher-Goeppinger S. Probst H. C. Gentzel M. Wilm M. (2011). p38 MAPK controls prothrombin expression by regulated RNA 3′ end processing. Mol. Cell 41, 298–310 10.1016/j.molcel.2010.12.032 21292162

[B39] Danckwardt S. Gehring N. H. Neu-Yilik G. Hundsdoerfer P. Pforsich M. Frede U. (2004). The prothrombin 3′end formation signal reveals a unique architecture that is sensitive to thrombophilic gain-of-function mutations. Blood 104, 428–435 10.1182/blood-2003-08-2894 15059842

[B40] Danckwardt S. Hartmann K. Katz B. Hentze M. W. Levy Y. Eichele R. (2006). The prothrombin 20209 C → T mutation in Jewish-Moroccan Caucasians: molecular analysis of gain-of-function of 3′ end processing. J. Thromb. Haemost. 4, 1078–1085 10.1111/j.1538-7836.2006.02126.x 16689762

[B41] Danckwardt S. Hentze M. W. Kulozik A. E. (2008). 3′ end mRNA processing: molecular mechanisms and implications for health and disease. EMBO J. 27, 482–498 10.1038/sj.emboj.7601932 18256699 PMC2241648

[B42] Danckwardt S. Kaufmann I. Gentzel M. Foerstner K. U. Gantzert A.-S. Gehring N. H. (2007). Splicing factors stimulate polyadenylation via USEs at non-canonical 3′ end formation signals. EMBO J. 26, 2658–2669 10.1038/sj.emboj.7601699 17464285 PMC1888663

[B43] Darmon S. K. Lutz C. S. (2012). mRNA 3′ end processing factors: a phylogenetic comparison. Comp. Funct. Genomics 2012, 876893 10.1155/2012/876893 22400011 PMC3287031

[B44] Dass B. Attaya E. N. Michelle Wallace A. MacDonald C. C. (2001). Overexpression of the CstF-64 and CPSF-160 polyadenylation protein messenger RNAs in mouse male germ cells. Biol. Reprod. 64, 1722–1729 10.1095/biolreprod64.6.1722 11369601

[B45] Dass B. Tardif S. Park J. Y. Tian B. Weitlauf H. M. Hess R. A. (2007). Loss of polyadenylation protein tauCstF-64 causes spermatogenic defects and male infertility. Proc. Natl. Acad. Sci. U.S.A. 104, 20374–20379 10.1073/pnas.0707589104 18077340 PMC2154438

[B46] de Klerk E. Venema A. Anvar S. Y. Goeman J. J. Hu O. Trollet C. (2012). Poly(A) binding protein nuclear 1 levels affect alternative polyadenylation. Nucleic Acids Res. 40, 9089–9101 10.1093/nar/gks655 22772983 PMC3467053

[B47] Derti A. Garrett-Engele P. Macisaac K. D. Stevens R. C. Sriram S. Chen R. (2012). A quantitative atlas of polyadenylation in five mammals. Genome Res. 22, 1173–1183 10.1101/gr.132563.111 22454233 PMC3371698

[B48] Di Giammartino D. C. Nishida K. Manley J. L. (2011). Mechanisms and consequences of alternative polyadenylation. Mol. Cell 43, 853–866 10.1016/j.molcel.2011.08.017 21925375 PMC3194005

[B49] Di Giammartino D. C. Shi Y. Manley J. L. (2013). PARP1 represses PAP and inhibits polyadenylation during heat shock. Mol. Cell 49, 7–17 23219533 10.1016/j.molcel.2012.11.005 PMC3545032

[B50] Dresser D. W. Hacker A. Lovell-Badge R. Guerrier D. (1995). The genes for a spliceosome protein (SAP62) and the anti-Müllerian hormone (AMH) are contiguous. Hum. Mol. Genet. 4, 1613–1618 10.1093/hmg/4.9.1613 8541848

[B51] Duan H. Cherradi N. Feige J.-J. Jefcoate C. (2009). cAMP-dependent posttranscriptional regulation of steroidogenic acute regulatory (STAR) protein by the zinc finger protein ZFP36L1/TIS11b. Mol. Endocrinol. 23, 497–509 10.1210/me.2008-0296 19179481 PMC2667709

[B52] Elkon R. Drost J. Van Haaften G. Jenal M. Schrier M. Vrielink J. A. O. (2012). E2F mediates enhanced alternative polyadenylation in proliferation. Genome Biol. 13, R59 10.1186/gb-2012-13-7-r59 22747694 PMC3491381

[B53] Evsyukova I. Bradrick S. S. Gregory S. G. Garcia-Blanco M. A. (2012). Cleavage and polyadenylation specificity factor 1 (CPSF1) regulates alternative splicing of interleukin 7 receptor (IL7R) exon 6. RNA 19, 103–115 10.1261/rna.035410.112 23151878 PMC3527722

[B54] Flaherty S. M. Fortes P. Izaurralde E. Mattaj I. W. Gilmartin G. M. (1997). Participation of the nuclear cap binding complex in pre-mRNA 3′ processing. Proc. Natl. Acad. Sci. U.S.A. 94, 11893–11898 10.1073/pnas.94.22.11893 9342333 PMC23648

[B55] Flavell S. W. Kim T.-K. Gray J. M. Harmin D. A. Hemberg M. Hong E. J. (2008). Genome-wide analysis of MEF2 transcriptional program reveals synaptic target genes and neuronal activity-dependent polyadenylation site selection. Neuron 60, 1022–1038 10.1016/j.neuron.2008.11.029 19109909 PMC2630178

[B56] Fortes P. Cuevas Y. Guan F. Liu P. Pentlicky S. Jung S. P. (2003). Inhibiting expression of specific genes in mammalian cells with 5′ end-mutated U1 small nuclear RNAs targeted to terminal exons of pre-mRNA. Proc. Natl. Acad. Sci. U.S.A. 100, 8264–8269 10.1073/pnas.1332669100 12826613 PMC166217

[B57] Foulkes N. S. Schlotter F. Pévet P. Sassone-Corsi P. (1993). Pituitary hormone FSH directs the CREM functional switch during spermatogenesis. Nature 362, 264–267 10.1038/362264a0 7681549

[B58] Fox-Walsh K. Davis-Turak J. Zhou Y. Li H. Fu X.-D. (2011). A multiplex RNA-seq strategy to profile poly(A+) RNA: application to analysis of transcription response and 3′ end formation. Genomics 98, 266–271 10.1016/j.ygeno.2011.04.003 21515359 PMC3160523

[B59] Friedman R. C. Farh K. K.-H. Burge C. B. Bartel D. P. (2009). Most mammalian mRNAs are conserved targets of microRNAs. Genome Res. 19, 92–105 10.1101/gr.082701.108 18955434 PMC2612969

[B60] Fu Y. Sun Y. Li Y. Li J. Rao X. Chen C. (2011). Differential genome-wide profiling of tandem 3′ UTRs among human breast cancer and normal cells by high-throughput sequencing. Genome Res. 21, 741–747 10.1101/gr.115295.110 21474764 PMC3083091

[B61] Garin I. Edghill E. L. Akerman I. Rubio-Cabezas O. Rica I. Locke J. M. (2010). Recessive mutations in the INS gene result in neonatal diabetes through reduced insulin biosynthesis. Proc. Natl. Acad. Sci. U.S.A. 107, 3105–3110 10.1073/pnas.0910533107 20133622 PMC2840338

[B62] Gartner W. Mineva I. Daneva T. Baumgartner-Parzer S. Niederle B. Vierhapper H. (2005). A newly identified RET proto-oncogene polymorphism is found in a high number of endocrine tumor patients. Hum. Genet. 117, 143–153 10.1007/s00439-005-1280-5 15841388

[B63] Gee A. H. Kasprzak W. Shapiro B. A. (2006). Structural differentiation of the HIV-1 polyA signals. J. Biomol. Struct. Dyn. 23, 417–428 10.1080/07391102.2006.10531236 16363877

[B64] Gehring N. H. Frede U. Neu-Yilik G. Hundsdoerfer P. Vetter B. Hentze M. W. (2001). Increased efficiency of mRNA 3′ end formation: a new genetic mechanism contributing to hereditary thrombophilia. Nat. Genet. 28, 389–392 10.1038/ng578 11443298

[B65] Gieselmann V. Polten A. Kreysing J. Von Figura K. (1989). Arylsulfatase A pseudodeficiency: loss of a polyadenylylation signal and N-glycosylation site. Proc. Natl. Acad. Sci. U.S.A. 86, 9436–9440 10.1073/pnas.86.23.9436 2574462 PMC298511

[B66] Goraczniak R. Behlke M. A. Gunderson S. I. (2009). Gene silencing by synthetic U1 adaptors. Nat. Biotechnol. 27, 257–263 10.1038/nbt.1525 19219028

[B67] Gotlib J. Cools J. (2008). Five years since the discovery of FIP1L1-PDGFRA: what we have learned about the fusion and other molecularly defined eosinophilias. Leukemia 22, 1999–2010 10.1038/leu.2008.287 18843283

[B68] Graber J. H. McAllister G. D. Smith T. F. (2002). Probabilistic prediction of *Saccharomyces cerevisiae* mRNA 3′-processing sites. Nucleic Acids Res. 30, 1851–1858 10.1093/nar/30.8.1851 11937640 PMC113205

[B69] Gruber A. R. Martin G. Keller W. Zavolan M. (2012). Cleavage Factor Im is a key regulator of 3′ UTR length. RNA Biol. 9, 1405–1412 10.4161/rna.22570 23187700

[B70] Gunderson S. I. Polycarpou-Schwarz M. Mattaj I. W. (1998). U1 snRNP inhibits pre-mRNA polyadenylation through a direct interaction between U1 70K and poly(A) polymerase. Mol. Cell 1, 255–264 10.1016/S1097-2765(00)80026-X 9659922

[B71] Gunderson S. I. Vagner S. Polycarpou-Schwarz M. Mattaj I. W. (1997). Involvement of the carboxyl terminus of vertebrate poly(A) polymerase in U1A autoregulation and in the coupling of splicing and polyadenylation. Genes Dev. 11, 761–773 10.1101/gad.11.6.761 9087430

[B72] Guo J. Garrett M. Micklem G. Brogna S. (2011). Poly(A) signals located near the 5′ end of genes are silenced by a general mechanism that prevents premature 3′-end processing. Mol. Cell. Biol. 31, 639–651 10.1128/MCB.00919-10 21135120 PMC3028650

[B73] Guo S. S. Wu X. Shimoide A. T. Wong J. Moatamed F. Sawicki M. P. (2003). Frequent overexpression of cyclin D1 in sporadic pancreatic endocrine tumours. J. Endocrinol. 179, 73–79 10.1677/joe.0.1790073 14529567

[B74] Gyawali S. Subaran R. Weissman M. M. Hershkowitz D. McKenna M. C. Talati A. (2010). Association of a polyadenylation polymorphism in the serotonin transporter and panic disorder. Biol. Psychiatry 67, 331–338 10.1016/j.biopsych.2009.10.015 19969287 PMC2980348

[B75] Hajarnavis A. Korf I. Durbin R. (2004). A probabilistic model of 3′ end formation in *Caenorhabditis elegans*. Nucleic Acids Res. 32, 3392–3399 10.1093/nar/gkh656 15247332 PMC443532

[B76] Hall-Pogar T. Liang S. Hague L. K. Lutz C. S. (2007). Specific trans-acting proteins interact with auxiliary RNA polyadenylation elements in the COX-2 3′-UTR. RNA 13, 1103–1115 10.1261/rna.577707 17507659 PMC1894925

[B77] Harteveld C. L. Losekoot M. Haak H. Heister G. A. Giordano P. C. Bernini L. F. (1994). A novel polyadenylation signal mutation in the alpha 2-globin gene causing alpha thalassaemia. Br. J. Haematol. 87, 139–143 10.1111/j.1365-2141.1994.tb04883.x 7947237

[B78] Hartley C. A. McKenna M. C. Salman R. Holmes A. Casey B. J. Phelps E. A. (2012). Serotonin transporter polyadenylation polymorphism modulates the retention of fear extinction memory. Proc. Natl. Acad. Sci. U.S.A. 109, 5493–5498 10.1073/pnas.1202044109 22431634 PMC3325655

[B79] Heisler L. K. Pronchuk N. Nonogaki K. Zhou L. Raber J. Tung L. (2007). Serotonin activates the hypothalamic-pituitary-adrenal axis via serotonin 2C receptor stimulation. J. Neurosci. 27, 6956–6964 10.1523/JNEUROSCI.2584-06.2007 17596444 PMC6672238

[B80] Hellquist A. Zucchelli M. Kivinen K. Saarialho-Kere U. Koskenmies S. Widen E. (2007). The human GIMAP5 gene has a common polyadenylation polymorphism increasing risk to systemic lupus erythematosus. J. Med. Genet. 44, 314–321 10.1136/jmg.2006.046185 17220214 PMC2597989

[B81] Higgs D. R. Goodbourn S. E. Lamb J. Clegg J. B. Weatherall D. J. Proudfoot N. J. (1983). Alpha-thalassaemia caused by a polyadenylation signal mutation. Nature 306, 398–400 10.1038/306398a0 6646217

[B82] Hilgers V. Perry M. W. Hendrix D. Stark A. Levine M. Haley B. (2011). Neural-specific elongation of 3′ UTRs during Drosophila development. Proc. Natl. Acad. Sci. U.S.A. 108, 15864–15869 10.1073/pnas.1112672108 21896737 PMC3179109

[B83] Ho E. S. Gunderson S. I. (2011). Long conserved fragments upstream of Mammalian polyadenylation sites. Genome Biol. Evol. 3, 654–666 10.1093/gbe/evr053 21705472 PMC3157836

[B84] Hockert K. J. Martincic K. Mendis-Handagama S. M. L. C. Borghesi L. A. Milcarek C. Dass B. (2011). Spermatogenetic but not immunological defects in mice lacking the τCstF-64 polyadenylation protein. J. Reprod. Immunol. 89, 26–37 10.1016/j.jri.2011.01.018 21489638 PMC3081895

[B85] Hogg J. R. Goff S. P. (2010). Upf1 senses 3′UTR length to potentiate mRNA decay. Cell 143, 379–389 10.1016/j.cell.2010.10.005 21029861 PMC2981159

[B86] Hoque M. Ji Z. Zheng D. Luo W. Li W. You B. (2013). Analysis of alternative cleavage and polyadenylation by 3′ region extraction and deep sequencing. Nat. Methods 10, 133–139 10.1038/nmeth.2288 23241633 PMC3560312

[B87] Hung L.-H. Heiner M. Hui J. Schreiner S. Benes V. Bindereif A. (2008). Diverse roles of hnRNP L in mammalian mRNA processing: a combined microarray and RNAi analysis. RNA 14, 284–296 10.1261/rna.725208 18073345 PMC2212255

[B88] Imesch P. Hornung R. Fink D. Fedier A. (2011). Cordycepin (3′-deoxyadenosine), an inhibitor of mRNA polyadenylation, suppresses proliferation and activates apoptosis in human epithelial endometriotic cells in vitro. Gynecol. Obstet. Invest. 72, 43–49 10.1159/000322395 21196698

[B89] Jan C. H. Friedman R. C. Ruby J. G. Bartel D. P. (2011). Formation, regulation and evolution of *Caenorhabditis elegans* 3′UTRs. Nature 469, 97–101 10.1038/nature09616 21085120 PMC3057491

[B90] Jankovic L. Efremov G. D. Petkov G. Kattamis C. George E. Yang K. G. (1990). Two novel polyadenylation mutations leading to beta(+)-thalassemia. Br. J. Haematol. 75, 122–126 10.1111/j.1365-2141.1990.00122.x 2375910

[B91] Jenal M. Elkon R. Loayza-Puch F. Van Haaften G. Kühn U. Menzies F. M. (2012). The poly(A)-binding protein nuclear 1 suppresses alternative cleavage and polyadenylation sites. Cell 149, 538–553 10.1016/j.cell.2012.03.022 22502866

[B92] Ji Z. Lee J. Y. Pan Z. Jiang B. Tian B. (2009). Progressive lengthening of 3′ untranslated regions of mRNAs by alternative polyadenylation during mouse embryonic development. Proc. Natl. Acad. Sci. U.S.A. 106, 7028–7033 10.1073/pnas.0900028106 19372383 PMC2669788

[B93] Ji Z. Luo W. Li W. Hoque M. Pan Z. Zhao Y. (2011). Transcriptional activity regulates alternative cleavage and polyadenylation. Mol. Syst. Biol. 7, 534 10.1038/msb.2011.69 21952137 PMC3202805

[B94] Ji Z. Tian B. (2009). Reprogramming of 3′ untranslated regions of mRNAs by alternative polyadenylation in generation of pluripotent stem cells from different cell types. PLoS ONE 4:e8419 10.1371/journal.pone.0008419 20037631 PMC2791866

[B95] Jonkers Y. M. H. Claessen S. M. H. Perren A. Schmitt A. M. Hofland L. J. De Herder W. (2007). DNA copy number status is a powerful predictor of poor survival in endocrine pancreatic tumor patients. Endocr. Relat. Cancer 14, 769–779 10.1677/ERC-07-0111 17914106

[B96] Kaida D. Berg M. G. Younis I. Kasim M. Singh L. N. Wan L. (2010). U1 snRNP protects pre-mRNAs from premature cleavage and polyadenylation. Nature 468, 664–668 10.1038/nature09479 20881964 PMC2996489

[B97] Keene J. D. (2007). RNA regulons: coordination of post-transcriptional events. Nat. Rev. Genet. 8, 533–543 10.1038/nrg2111 17572691

[B98] Khaladkar M. Smyda M. Hannenhalli S. (2011). Epigenomic and RNA structural correlates of polyadenylation. RNA Biol. 8, 529–537 10.4161/rna.8.3.15194 21508683 PMC3218514

[B99] Kiefer H. Mizutani A. Iemura S.-I. Natsume T. Ando H. Kuroda Y. (2009). Inositol 1,4,5-triphosphate receptor-binding protein released with inositol 1,4,5-triphosphate (IRBIT) associates with components of the mRNA 3′ processing machinery in a phosphorylation-dependent manner and inhibits polyadenylation. J. Biol. Chem. 284, 10694–10705 10.1074/jbc.M807136200 19224921 PMC2667756

[B100] Kim H. Lee J. H. Lee Y. (2003). Regulation of poly(A) polymerase by 14-3-3epsilon. EMBO J. 22, 5208–5219 10.1093/emboj/cdg497 14517258 PMC204469

[B101] Kim S. Yamamoto J. Chen Y. Aida M. Wada T. Handa H. (2010). Evidence that cleavage factor Im is a heterotetrameric protein complex controlling alternative polyadenylation. Genes Cells 15, 1003–1013 10.1111/j.1365-2443.2010.01436.x 20695905

[B102] Kleiman F. E. Manley J. L. (2001). The BARD1-CstF-50 interaction links mRNA 3′ end formation to DNA damage and tumor suppression. Cell 104, 743–753 10.1016/S0092-8674(01)00270-7 11257228

[B103] Ko B. Gunderson S. I. (2002). Identification of new poly(A) polymerase-inhibitory proteins capable of regulating pre-mRNA polyadenylation. J. Mol. Biol. 318, 1189–1206 10.1016/S0022-2836(02)00240-1 12083511

[B104] Kondrashov A. Meijer H. A. Barthet-Barateig A. Parker H. N. Khurshid A. Tessier S. (2012). Inhibition of polyadenylation reduces inflammatory gene induction. RNA 18, 2236–2250 10.1261/rna.032391.112 23118416 PMC3504674

[B105] Kubo T. Wada T. Yamaguchi Y. Shimizu A. Handa H. (2006). Knock-down of 25 kDa subunit of cleavage factor Im in Hela cells alters alternative polyadenylation within 3′-UTRs. Nucleic Acids Res. 34, 6264–6271 10.1093/nar/gkl794 17098938 PMC1669743

[B106] Kühn U. Gündel M. Knoth A. Kerwitz Y. Rüdel S. Wahle E. (2009). Poly(A) tail length is controlled by the nuclear poly(A)-binding protein regulating the interaction between poly(A) polymerase and the cleavage and polyadenylation specificity factor. J. Biol. Chem. 284, 22803–22814 10.1074/jbc.M109.037614 19509282 PMC2755688

[B107] Kwan T. Benovoy D. Dias C. Gurd S. Provencher C. Beaulieu P. (2008). Genome-wide analysis of transcript isoform variation in humans. Nat. Genet. 40, 225–231 10.1038/ng.2007.57 18193047

[B108] Kyburz A. Friedlein A. Langen H. Keller W. (2006). Direct interactions between subunits of CPSF and the U2 snRNP contribute to the coupling of pre-mRNA 3′ end processing and splicing. Mol. Cell 23, 195–205 10.1016/j.molcel.2006.05.037 16857586

[B109] Laishram R. S. Barlow C. A. Anderson R. A. (2011). CKI isoforms α and ε regulate Star-PAP target messages by controlling Star-PAP poly(A) polymerase activity and phosphoinositide stimulation. Nucleic Acids Res. 39, 7961–7973 10.1093/nar/gkr549 21729869 PMC3185439

[B110] Lee E. K. Gorospe M. (2010). Minireview: posttranscriptional regulation of the insulin and insulin-like growth factor systems. Endocrinology 151, 1403–1408 10.1210/en.2009-1123 20032049 PMC2850238

[B111] Lee Y. J. Lee Y. Chung J. H. (2000). An intronless gene encoding a poly(A) polymerase is specifically expressed in testis. FEBS Lett. 487, 287–292 10.1016/S0014-5793(00)02367-X 11150526

[B112] Legendre M. Gautheret D. (2003). Sequence determinants in human polyadenylation site selection. BMC Genomics 4:7 10.1186/1471-2164-4-7 12600277 PMC151664

[B113] Legendre M. Ritchie W. Lopez F. Gautheret D. (2006). Differential repression of alternative transcripts: a screen for miRNA targets. PLoS Comput. Biol. 2:e43 10.1371/journal.pcbi.0020043 16699595 PMC1458965

[B114] Lejeune F. Maquat L. E. (2005). Mechanistic links between nonsense-mediated mRNA decay and pre-mRNA splicing in mammalian cells. Curr. Opin. Cell Biol. 17, 309–315 10.1016/j.ceb.2005.03.002 15901502

[B115] Lembo A. Di Cunto F. Provero P. (2012). Shortening of 3′UTRs correlates with poor prognosis in breast and lung cancer. PLoS ONE 7:e31129 10.1371/journal.pone.0031129 22347440 PMC3275581

[B116] Levy J. R. Hannah S. Mooney R. L. Hug V. Stevens W. (1995). Sequence and functional characterization of the terminal exon of the human insulin receptor gene. Biochim. Biophys. Acta 1263, 253–257 10.1016/0167-4781(95)00107-R 7548214

[B117] Li B. Zhang G. Li C. He D. Li X. Zhang C. (2012a). Identification of JAK2 as a mediator of FIP1L1-PDGFRA-induced eosinophil growth and function in CEL. PLoS ONE 7:e34912 10.1371/journal.pone.0034912 22523564 PMC3327703

[B118] Li W. Laishram R. S. Ji Z. Barlow C. A. Tian B. Anderson R. A. (2012b). Star-PAP control of BIK expression and apoptosis is regulated by nuclear PIPKIα and PKCδ signaling. Mol. Cell 45, 25–37 10.1016/j.molcel.2011.11.017 22244330 PMC3268557

[B119] Li W. Yeh H.-J. Shankarling G. S. Ji Z. Tian B. Macdonald C. C. (2012c). The τCstF-64 polyadenylation protein controls genome expression in testis. PLoS ONE 7:e48373 10.1371/journal.pone.0048373 23110235 PMC3482194

[B120] Lian Z. Karpikov A. Lian J. Mahajan M. C. Hartman S. Gerstein M. (2008). A genomic analysis of RNA polymerase II modification and chromatin architecture related to 3′ end RNA polyadenylation. Genome Res. 18, 1224–1237 10.1101/gr.075804.107 18487515 PMC2493437

[B121] Licatalosi D. D. Geiger G. Minet M. Schroeder S. Cilli K. McNeil J. B. (2002). Functional interaction of yeast pre-mRNA 3′ end processing factors with RNA polymerase II. Mol. Cell 9, 1101–1111 10.1016/S1097-2765(02)00518-X 12049745

[B122] Licatalosi D. D. Mele A. Fak J. J. Ule J. Kayikci M. Chi S. W. (2008). HITS-CLIP yields genome-wide insights into brain alternative RNA processing. Nature 456, 464–469 10.1038/nature07488 18978773 PMC2597294

[B123] Lin Y. Li Z. Ozsolak F. Kim S. W. Arango-Argoty G. Liu T. T. (2012). An in-depth map of polyadenylation sites in cancer. Nucleic Acids Res. 40, 8460–8471 10.1093/nar/gkr1078 22753024 PMC3458571

[B124] Lisman T. De Groot P. G. Meijers J. C. M. Rosendaal F. R. (2005). Reduced plasma fibrinolytic potential is a risk factor for venous thrombosis. Blood 105, 1102–1105 10.1182/blood-2004-08-3253 15466929

[B125] Liu D. Brockman J. M. Dass B. Hutchins L. N. Singh P. McCarrey J. R. (2007). Systematic variation in mRNA 3′-processing signals during mouse spermatogenesis. Nucleic Acids Res. 35, 234–246 10.1093/nar/gkm859 17158511 PMC1802579

[B126] Liu D. Waxman D. J. (2002). Post-transcriptional regulation of hepatic NADPH-cytochrome P450 reductase by thyroid hormone: independent effects on poly(A) tail length and mRNA stability. Mol. Pharmacol. 61, 1089–1096 10.1124/mol.61.5.1089 11961126

[B127] Locke J. M. Da Silva Xavier G. Rutter G. A. Harries L. W. (2011). An alternative polyadenylation signal in TCF7L2 generates isoforms that inhibit T cell factor/lymphoid-enhancer factor (TCF/LEF)-dependent target genes. Diabetologia 54, 3078–3082 10.1007/s00125-011-2327-x 21913056 PMC3210366

[B128] Lou H. Neugebauer K. M. Gagel R. F. Berget S. M. (1998). Regulation of alternative polyadenylation by U1 snRNPs and SRp20. Mol. Cell. Biol. 18, 4977–4985 9710581 10.1128/mcb.18.9.4977 PMC109082

[B129] Luna R. Jimeno S. Marín M. Huertas P. García-Rubio M. Aguilera A. (2005). Interdependence between transcription and mRNP processing and export, and its impact on genetic stability. Mol. Cell 18, 711–722 10.1016/j.molcel.2005.05.001 15949445

[B130] Lunde B. M. Reichow S. L. Kim M. Suh H. Leeper T. C. Yang F. (2010). Cooperative interaction of transcription termination factors with the RNA polymerase II C-terminal domain. Nat. Struct. Mol. Biol. 17, 1195–1201 10.1038/nsmb.1893 20818393 PMC2950884

[B131] MacDonald C. C. Wilusz J. Shenk T. (1994). The 64-kilodalton subunit of the CstF polyadenylation factor binds to pre-mRNAs downstream of the cleavage site and influences cleavage site location. Mol. Cell. Biol. 14, 6647–6654 7935383 10.1128/mcb.14.10.6647-6654.1994 PMC359194

[B132] MacIsaac J. L. Bogutz A. B. Morrissy A. S. Lefebvre L. (2012). Tissue-specific alternative polyadenylation at the imprinted gene Mest regulates allelic usage at Copg2. Nucleic Acids Res. 40, 1523–1535 10.1093/nar/gkr871 22053079 PMC3287194

[B133] Mandel C. R. Kaneko S. Zhang H. Gebauer D. Vethantham V. Manley J. L. (2006). Polyadenylation factor CPSF-73 is the pre-mRNA 3′-end-processing endonuclease. Nature 444, 953–956 10.1038/nature05363 17128255 PMC3866582

[B134] Mangone M. Manoharan A. P. Thierry-Mieg D. Thierry-Mieg J. Han T. Mackowiak S. D. (2010). The landscape of *C. elegans* 3′UTRs. Science 329, 432–435 10.1126/science.1191244 20522740 PMC3142571

[B135] Mapendano C. K. Lykke-Andersen S. Kjems J. Bertrand E. Jensen T. H. (2010). Crosstalk between mRNA 3′ end processing and transcription initiation. Mol. Cell 40, 410–422 10.1016/j.molcel.2010.10.012 21070967

[B136] Martin G. Gruber A. R. Keller W. Zavolan M. (2012). Genome-wide analysis of pre-mRNA 3′ end processing reveals a decisive role of human cleavage factor I in the regulation of 3′ UTR length. Cell Rep. 1, 753–763 10.1016/j.celrep.2012.05.003 22813749

[B137] Martincic K. Alkan S. A. Cheatle A. Borghesi L. Milcarek C. (2009). Transcription elongation factor ELL2 directs immunoglobulin secretion in plasma cells by stimulating altered RNA processing. Nat. Immunol. 10, 1102–1109 10.1038/ni.1786 19749764 PMC2771556

[B138] Martincic K. Campbell R. Edwalds-Gilbert G. Souan L. Lotze M. T. Milcarek C. (1998). Increase in the 64-kDa subunit of the polyadenylation/cleavage stimulatory factor during the G0 to S phase transition. Proc. Natl. Acad. Sci. U.S.A. 95, 11095–11100 10.1073/pnas.95.19.11095 9736695 PMC21601

[B139] Mayr C. Bartel D. P. (2009). Widespread shortening of 3′UTRs by alternative cleavage and polyadenylation activates oncogenes in cancer cells. Cell 138, 673–684 10.1016/j.cell.2009.06.016 19703394 PMC2819821

[B140] McCracken S. Fong N. Yankulov K. Ballantyne S. Pan G. Greenblatt J. (1997). The C-terminal domain of RNA polymerase II couples mRNA processing to transcription. Nature 385, 357–361 10.1038/385357a0 9002523

[B141] McCracken S. Lambermon M. Blencowe B. J. (2002). SRm160 splicing coactivator promotes transcript 3′-end cleavage. Mol. Cell. Biol. 22, 148–160 10.1128/MCB.22.1.148-160.2002 11739730 PMC134228

[B142] McCracken S. Longman D. Johnstone I. L. Cáceres J. F. Blencowe B. J. (2003). An evolutionarily conserved role for SRm160 in 3′-end processing that functions independently of exon junction complex formation. J. Biol. Chem. 278, 44153–44160 10.1074/jbc.M306856200 12944400

[B143] Mellman D. L. Gonzales M. L. Song C. Barlow C. A. Wang P. Kendziorski C. (2008). A PtdIns4,5P2-regulated nuclear poly(A) polymerase controls expression of select mRNAs. Nature 451, 1013–1017 10.1038/nature06666 18288197

[B144] Mercer T. R. Wilhelm D. Dinger M. E. Soldà G. Korbie D. J. Glazov E. A. (2011). Expression of distinct RNAs from 3′ untranslated regions. Nucleic Acids Res. 39, 2393–2403 10.1093/nar/gkq1158 21075793 PMC3064787

[B145] Millevoi S. Decorsière A. Loulergue C. Iacovoni J. Bernat S. Antoniou M. (2009). A physical and functional link between splicing factors promotes pre-mRNA 3′ end processing. Nucleic Acids Res. 37, 4672–4683 10.1093/nar/gkp470 19506027 PMC2724285

[B146] Millevoi S. Loulergue C. Dettwiler S. Karaa S. Z. Keller W. Antoniou M. (2006). An interaction between U2AF 65 and CF I(m) links the splicing and 3′ end processing machineries. EMBO J. 25, 4854–4864 10.1038/sj.emboj.7601331 17024186 PMC1618107

[B147] Millevoi S. Vagner S. (2010). Molecular mechanisms of eukaryotic pre-mRNA 3′ end processing regulation. Nucleic Acids Res. 38, 2757–2774 10.1093/nar/gkp1176 20044349 PMC2874999

[B148] Minvielle S. Giscard-Dartevelle S. Cohen R. Taboulet J. Labye F. Jullienne A. (1991). A novel calcitonin carboxyl-terminal peptide produced in medullary thyroid carcinoma by alternative RNA processing of the calcitonin/calcitonin gene-related peptide gene. J. Biol. Chem. 266, 24627–24631 1761559

[B149] Moreira A. Takagaki Y. Brackenridge S. Wollerton M. Manley J. L. Proudfoot N. J. (1998). The upstream sequence element of the C2 complement poly(A) signal activates mRNA 3′ end formation by two distinct mechanisms. Genes Dev. 12, 2522–2534 10.1101/gad.12.16.2522 9716405 PMC317083

[B150] Morris A. R. Bos A. Diosdado B. Rooijers K. Elkon R. Bolijn A. S. (2012). alternative cleavage and polyadenylation during colorectal cancer development. Clin. Cancer Res. 18, 5256–5266 10.1158/1078-0432.CCR-12-0543 22874640

[B151] Murthy K. G. Manley J. L. (1995). The 160-kD subunit of human cleavage-polyadenylation specificity factor coordinates pre-mRNA 3′-end formation. Genes Dev. 9, 2672–2683 10.1101/gad.9.21.2672 7590244

[B152] Nagaike T. Logan C. Hotta I. Rozenblatt-Rosen O. Meyerson M. Manley J. L. (2011). Transcriptional activators enhance polyadenylation of mRNA precursors. Mol. Cell 41, 409–418 10.1016/j.molcel.2011.01.022 21329879 PMC3060669

[B153] Nagamatsu T. Fujii T. Kusumi M. Zou L. Yamashita T. Osuga Y. (2004). Cytotrophoblasts up-regulate soluble fms-like tyrosine kinase-1 expression under reduced oxygen: an implication for the placental vascular development and the pathophysiology of preeclampsia. Endocrinology 145, 4838–4845 10.1210/en.2004-0533 15284201

[B154] Nandy D. Mukhopadhyay D. Basu A. (2010). Both vascular endothelial growth factor and soluble Flt-1 are increased in type 2 diabetes but not in impaired fasting glucose. J. Investig. Med. 58, 804–806 20571438 10.231/JIM.0b013e3181e96203 PMC3677544

[B155] Nazeer F. I. Devany E. Mohammed S. Fonseca D. Akukwe B. Taveras C. (2011). p53 inhibits mRNA 3′ processing through its interaction with the CstF/BARD1 complex. Oncogene 30, 3073–3083 10.1038/onc.2011.29 21383700

[B156] Neilson J. R. Sandberg R. (2010). Heterogeneity in mammalian RNA 3′ end formation. Exp. Cell Res. 316, 1357–1364 10.1016/j.yexcr.2010.02.040 20211174 PMC2866830

[B157] Nemeroff M. E. Barabino S. M. Li Y. Keller W. Krug R. M. (1998). Influenza virus NS1 protein interacts with the cellular 30 kDa subunit of CPSF and inhibits 3′end formation of cellular pre-mRNAs. Mol. Cell 1, 991–1000 10.1016/S1097-2765(00)80099-4 9651582

[B158] Nunes N. M. Li W. Tian B. Furger A. (2010). A functional human Poly(A) site requires only a potent DSE and an A-rich upstream sequence. EMBO J. 29, 1523–1536 10.1038/emboj.2010.42 20339349 PMC2876958

[B159] Okuhara K. Abe S. Kondo T. Fujita K. Koda N. Mochizuki H. (2008). Four Japanese patients with adrenal hypoplasia congenita and hypogonadotropic hypogonadism caused by DAX-1 gene mutations: mutant DAX-1 failed to repress steroidogenic acute regulatory protein (StAR) and luteinizing hormone beta-subunit gene promoter acti. Endocr. J. 55, 97–103 10.1507/endocrj.K07E-008 18202527

[B160] Oppelt P. Strissel P. L. Kellermann A. Seeber S. Humeny A. Beckmann M. W. (2005). DNA sequence variations of the entire anti-Mullerian hormone (AMH) gene promoter and AMH protein expression in patients with the Mayer-Rokitanski-Kuster-Hauser syndrome. Hum. Reprod. 20, 149–157 10.1093/humrep/deh547 15550498

[B161] Orkin S. H. Cheng T. C. Antonarakis S. E. Kazazian H. H. (1985). Thalassemia due to a mutation in the cleavage-polyadenylation signal of the human beta-globin gene. EMBO J. 4, 453–456 4018033 10.1002/j.1460-2075.1985.tb03650.x PMC554207

[B162] Ozsolak F. Kapranov P. Foissac S. Kim S. W. Fishilevich E. Monaghan A. P. (2010). Comprehensive polyadenylation site maps in yeast and human reveal pervasive alternative polyadenylation. Cell 143, 1018–1029 10.1016/j.cell.2010.11.020 21145465 PMC3022516

[B163] Parks L. L. Turney M. K. Detera-Wadleigh S. Kovacs W. J. (1998). An ACTH-producing small cell lung cancer expresses aberrant glucocorticoid receptor transcripts from a normal gene. Mol. Cell. Endocrinol. 142, 175–181 10.1016/S0303-7207(98)00107-5 9783913

[B164] Pauws E. Van Kampen A. H. Van de Graaf S. A. De Vijlder J. J. Ris-Stalpers C. (2001). Heterogeneity in polyadenylation cleavage sites in mammalian mRNA sequences: implications for SAGE analysis. Nucleic Acids Res. 29, 1690–1694 10.1093/nar/29.8.1690 11292841 PMC31324

[B165] Pelechano V. Wilkening S. Järvelin A. I. Tekkedil M. M. Steinmetz L. M. (2012). Genome-wide polyadenylation site mapping. Meth. Enzymol. 513, 271–296 10.1016/B978-0-12-391938-0.00012-4 22929774

[B166] Phillips C. Pachikara N. Gunderson S. I. (2004). U1A inhibits cleavage at the immunoglobulin M heavy-chain secretory poly(A) site by binding between the two downstream GU-rich regions. Mol. Cell. Biol. 24, 6162–6171 10.1128/MCB.24.14.6162-6171.2004 15226420 PMC434241

[B167] Pinto P. A. B. Henriques T. Freitas M. O. Martins T. Domingues R. G. Wyrzykowska P. S. (2011). RNA polymerase II kinetics in polo polyadenylation signal selection. EMBO J. 30, 2431–2444 10.1038/emboj.2011.156 21602789 PMC3116286

[B168] Proudfoot N. J. (2011). Ending the message: poly(A) signals then and now. Genes Dev. 25, 1770–1782 10.1101/gad.17268411 21896654 PMC3175714

[B169] Ptitsyn A. A. Gimble J. M. (2007). Analysis of circadian pattern reveals tissue-specific alternative transcription in leptin signaling pathway. BMC Bioinformatics 8(Suppl. 7):S15 10.1186/1471-2105-8-S7-S15 18047714 PMC2099483

[B170] Retelska D. Iseli C. Bucher P. Jongeneel C. V. Naef F. (2006). Similarities and differences of polyadenylation signals in human and fly. BMC Genomics 7:176 10.1186/1471-2164-7-176 16836751 PMC1574307

[B171] Rozenblatt-Rosen O. Nagaike T. Francis J. M. Kaneko S. Glatt K. A. Hughes C. M. (2009). The tumor suppressor Cdc73 functionally associates with CPSF and CstF 3′ mRNA processing factors. Proc. Natl. Acad. Sci. U.S.A. 106, 755–760 10.1073/pnas.0812023106 19136632 PMC2615665

[B172] Rund D. Dowling C. Najjar K. Rachmilewitz E. A. Kazazian H. H. Oppenheim A. (1992). Two mutations in the beta-globin polyadenylylation signal reveal extended transcripts and new RNA polyadenylylation sites. Proc. Natl. Acad. Sci. U.S.A. 89, 4324–4328 10.1073/pnas.89.10.4324 1374896 PMC49074

[B173] Ryan K. Bauer D. L. V. (2008). Finishing touches: post-translational modification of protein factors involved in mammalian pre-mRNA 3′ end formation. Int. J. Biochem. Cell Biol. 40, 2384–2396 10.1016/j.biocel.2008.03.016 18468939 PMC2548416

[B174] Sahakitrungruang T. Soccio R. E. Lang-Muritano M. Walker J. M. Achermann J. C. Miller W. L. (2010). Clinical, genetic, and functional characterization of four patients carrying partial loss-of-function mutations in the steroidogenic acute regulatory protein (StAR). J. Clin. Endocrinol. Metab. 95, 3352–3359 10.1210/jc.2010-0437 20444910 PMC2928910

[B175] Sandberg R. Neilson J. R. Sarma A. Sharp P. A. Burge C. B. (2008). Proliferating cells express mRNAs with shortened 3′ untranslated regions and fewer microRNA target sites. Science 320, 1643–1647 10.1126/science.1155390 18566288 PMC2587246

[B176] Sartini B. L. Wang H. Wang W. Millette C. F. Kilpatrick D. L. (2008). Pre-messenger RNA cleavage factor I (CFIm): potential role in alternative polyadenylation during spermatogenesis. Biol. Reprod. 78, 472–482 10.1095/biolreprod.107.064774 18032416

[B177] Shankarling G. S. Coates P. W. Dass B. Macdonald C. C. (2009). A family of splice variants of CstF-64 expressed in vertebrate nervous systems. BMC Mol. Biol. 10:22 10.1186/1471-2199-10-22 19284619 PMC2660332

[B178] Sheets M. D. Ogg S. C. Wickens M. P. (1990). Point mutations in AAUAAA and the poly (A) addition site: effects on the accuracy and efficiency of cleavage and polyadenylation in vitro. Nucleic Acids Res. 18, 5799–5805 10.1093/nar/18.19.5799 2170946 PMC332317

[B179] Shell S. A. Hesse C. Morris S. M. Milcarek C. (2005). Elevated levels of the 64-kDa cleavage stimulatory factor (CstF-64) in lipopolysaccharide-stimulated macrophages influence gene expression and induce alternative poly(A) site selection. J. Biol. Chem. 280, 39950–39961 10.1074/jbc.M508848200 16207706

[B180] Shepard P. J. Choi E.-A. Lu J. Flanagan L. A. Hertel K. J. Shi Y. (2011). Complex and dynamic landscape of RNA polyadenylation revealed by PAS-Seq. RNA 17, 761–772 10.1261/rna.2581711 21343387 PMC3062186

[B181] Shi Y. Di Giammartino D. C. Taylor D. Sarkeshik A. Rice W. J. Yates J. R. (2009). Molecular architecture of the human pre-mRNA 3′ processing complex. Mol. Cell 33, 365–376 10.1016/j.molcel.2008.12.028 19217410 PMC2946185

[B182] Shibata E. Rajakumar A. Powers R. W. Larkin R. W. Gilmour C. Bodnar L. M. (2005). Soluble fms-like tyrosine kinase 1 is increased in preeclampsia but not in normotensive pregnancies with small-for-gestational-age neonates: relationship to circulating placental growth factor. J. Clin. Endocrinol. Metab. 90, 4895–4903 10.1210/jc.2004-1955 15886253

[B183] Shin J.-H. Janer M. McNeney B. Blay S. Deutsch K. Sanjeevi C. B. (2007). IA-2 autoantibodies in incident type I diabetes patients are associated with a polyadenylation signal polymorphism in GIMAP5. Genes Immun. 8, 503–512 10.1038/sj.gene.6364413 17641683

[B184] Singh P. Alley T. L. Wright S. M. Kamdar S. Schott W. Wilpan R. Y. (2009). Global changes in processing of mRNA 3′ untranslated regions characterize clinically distinct cancer subtypes. Cancer Res. 69, 9422–9430 10.1158/0008-5472.CAN-08-1808 19934316 PMC2794997

[B185] Spies N. Nielsen C. B. Padgett R. A. Burge C. B. (2009). Biased chromatin signatures around polyadenylation sites and exons. Mol. Cell 36, 245–254 10.1016/j.molcel.2009.10.008 19854133 PMC2786773

[B186] Stacey S. N. Sulem P. Jonasdottir A. Masson G. Gudmundsson J. Gudbjartsson D. F. (2011). A germline variant in the TP53 polyadenylation signal confers cancer susceptibility. Nat. Genet. 43, 1098–1103 10.1038/ng.926 21946351 PMC3263694

[B187] Stirling P. C. Chan Y. A. Minaker S. W. Aristizabal M. J. Barrett I. Sipahimalani P. (2012). R-loop-mediated genome instability in mRNA cleavage and polyadenylation mutants. Genes Dev. 26, 163–175 10.1101/gad.179721.111 22279048 PMC3273840

[B188] Tabaska J. E. Zhang M. Q. (1999). Detection of polyadenylation signals in human DNA sequences. Gene 231, 77–86 10.1016/S0378-1119(99)00104-3 10231571

[B189] Takagaki Y. Manley J. L. (1998). Levels of polyadenylation factor CstF-64 control IgM heavy chain mRNA accumulation and other events associated with B cell differentiation. Mol. Cell 2, 761–771 10.1016/S1097-2765(00)80291-9 9885564

[B190] Tassone F. De Rubeis S. Carosi C. La Fata G. Serpa G. Raske C. (2011). Differential usage of transcriptional start sites and polyadenylation sites in FMR1 premutation alleles. Nucleic Acids Res. 39, 6172–6185 10.1093/nar/gkr100 21478165 PMC3152321

[B191] Thomadaki H. Scorilas A. Tsiapalis C. M. Havredaki M. (2008). The role of cordycepin in cancer treatment via induction or inhibition of apoptosis: implication of polyadenylation in a cell type specific manner. Cancer Chemother. Pharmacol. 61, 251–265 10.1007/s00280-007-0533-5 17487491

[B192] Thomas C. P. Andrews J. I. Liu K. Z. (2007). Intronic polyadenylation signal sequences and alternate splicing generate human soluble Flt1 variants and regulate the abundance of soluble Flt1 in the placenta. FASEB J. 21, 3885–3895 10.1096/fj.07-8809com 17615362

[B193] Thomas F. Sætrom P. (2012). Single nucleotide polymorphisms can create alternative polyadenylation signals and affect gene expression through loss of microRNA-regulation. PLoS Comput. Biol. 8:e1002621 10.1371/journal.pcbi.1002621 22915998 PMC3420919

[B194] Thomsen S. Azzam G. Kaschula R. Williams L. S. Alonso C. R. (2010). Developmental RNA processing of 3′UTRs in Hox mRNAs as a context-dependent mechanism modulating visibility to microRNAs. Development 137, 2951–2960 10.1242/dev.047324 20667912

[B195] Tian B. Graber J. H. (2012). Signals for pre-mRNA cleavage and polyadenylation. Wiley Interdiscip. Rev. RNA 3, 385–396 10.1002/wrna.116 22012871 PMC4451228

[B196] Tian B. Hu J. Zhang H. Lutz C. S. (2005). A large-scale analysis of mRNA polyadenylation of human and mouse genes. Nucleic Acids Res. 33, 201–212 10.1093/nar/gki158 15647503 PMC546146

[B197] Tian B. Pan Z. Lee J. Y. (2007). Widespread mRNA polyadenylation events in introns indicate dynamic interplay between polyadenylation and splicing. Genome Res. 17, 156–165 10.1101/gr.5532707 17210931 PMC1781347

[B198] Toniolo D. Rizzolio F. (2007). X chromosome and ovarian failure. Semin. Reprod. Med. 25, 264–271 10.1055/s-2007-980220 17594607

[B199] Topalian S. L. Kaneko S. Gonzales M. I. Bond G. L. Ward Y. Manley J. L. (2001). Identification and functional characterization of neo-poly(A) polymerase, an RNA processing enzyme overexpressed in human tumors. Mol. Cell. Biol. 21, 5614–5623 10.1128/MCB.21.16.5614-5623.2001 11463842 PMC87282

[B200] Touriol C. Morillon A. Gensac M. C. Prats H. Prats A. C. (1999). Expression of human fibroblast growth factor 2 mRNA is post-transcriptionally controlled by a unique destabilizing element present in the 3′-untranslated region between alternative polyadenylation sites. J. Biol. Chem. 274, 21402–21408 10.1074/jbc.274.30.21402 10409702

[B201] Uitte de Willige S. Rietveld I. M. De Visser M. C. H. Vos H. L. Bertina R. M. (2007). Polymorphism 10034C>T is located in a region regulating polyadenylation of FGG transcripts and influences the fibrinogen gamma′/gammaA mRNA ratio. J. Thromb. Haemost. 5, 1243–1249 10.1111/j.1538-7836.2007.02566.x 17403086

[B202] Van Oers C. C. Bakker L. Baas P. D. (1994). The exon 4 poly(A) site of the human calcitonin/CGRP-I pre-mRNA is a weak site in vitro. Biochim. Biophys. Acta 1218, 55–63 10.1016/0167-4781(94)90100-7 8193165

[B203] van Solinge W. W. Lind B. Van Wijk R. Hart H. C. Kraaijenhagen R. J. (1996). Clinical expression of a rare beta-globin gene mutation co-inherited with haemoglobin E-disease. Eur. J. Clin. Chem. Clin. Biochem. 34, 949–954 8986398 10.1515/cclm.1996.34.12.949

[B204] Venkataraman K. Brown K. M. Gilmartin G. M. (2005). Analysis of a noncanonical poly(A) site reveals a tripartite mechanism for vertebrate poly(A) site recognition. Genes Dev. 19, 1315–1327 10.1101/gad.1298605 15937220 PMC1142555

[B205] Veraldi K. L. Arhin G. K. Martincic K. Chung-Ganster L. H. Wilusz J. Milcarek C. (2001). hnRNP F influences binding of a 64-kilodalton subunit of cleavage stimulation factor to mRNA precursors in mouse B cells. Mol. Cell. Biol. 21, 1228–1238 10.1128/MCB.21.4.1228-1238.2001 11158309 PMC99576

[B206] Vickers T. A. Crooke S. T. (2012). siRNAs targeted to certain polyadenylation sites promote specific, RISC-independent degradation of messenger RNAs. Nucleic Acids Res. 40, 6223–6234 10.1093/nar/gks239 22422842 PMC3401429

[B207] Vickers T. A. Sabripour M. Crooke S. T. (2011). U1 adaptors result in reduction of multiple pre-mRNA species principally by sequestering U1snRNP. Nucleic Acids Res. 39, e71 10.1093/nar/gkr150 21415007 PMC3105408

[B208] Vorlová S. Rocco G. Lefave C. V. Jodelka F. M. Hess K. Hastings M. L. (2011). Induction of antagonistic soluble decoy receptor tyrosine kinases by intronic polyA activation. Mol. Cell 43, 927–939 10.1016/j.molcel.2011.08.009 21925381 PMC3781938

[B209] Wahba L. Amon J. D. Koshland D. Vuica-Ross M. (2011). RNase H and multiple RNA biogenesis factors cooperate to prevent RNA:DNA hybrids from generating genome instability. Mol. Cell 44, 978–988 10.1016/j.molcel.2011.10.017 22195970 PMC3271842

[B210] Wang E. T. Sandberg R. Luo S. Khrebtukova I. Zhang L. Mayr C. (2008). Alternative isoform regulation in human tissue transcriptomes. Nature 456, 470–476 10.1038/nature07509 18978772 PMC2593745

[B211] Wang L. Dowell R. D. Yi R. (2013). Genome-wide maps of polyadenylation reveal dynamic mRNA 3′-end formation in mammalian cell lineages. RNA 19, 413–425 10.1261/rna.035360.112 23325109 PMC3677251

[B212] Wang Y. Fairley J. A. Roberts S. G. E. (2010). Phosphorylation of TFIIB links transcription initiation and termination. Curr. Biol. 20, 548–553 10.1016/j.cub.2009.12.022 20226668 PMC2849011

[B213] West S. Proudfoot N. J. (2008). Human Pcf11 enhances degradation of RNA polymerase II-associated nascent RNA and transcriptional termination. Nucleic Acids Res. 36, 905–914 10.1093/nar/gkm1112 18086705 PMC2241900

[B214] West S. Proudfoot N. J. (2009). Transcriptional termination enhances protein expression in human cells. Mol. Cell 33, 354–364 10.1016/j.molcel.2009.01.008 19217409 PMC2706331

[B215] Wiestner A. Tehrani M. Chiorazzi M. Wright G. Gibellini F. Nakayama K. (2007). Point mutations and genomic deletions in CCND1 create stable truncated cyclin D1 mRNAs that are associated with increased proliferation rate and shorter survival. Blood 109, 4599–4606 10.1182/blood-2006-08-039859 17299095 PMC1885523

[B216] Wilkening S. Pelechano V. Järvelin A. I. Tekkedil M. M. Anders S. Benes V. (2013). An efficient method for genome-wide polyadenylation site mapping and RNA quantification. Nucleic Acids Res. 41, e65 10.1093/nar/gks1249 23295673 PMC3597643

[B217] Winter J. Kunath M. Roepcke S. Krause S. Schneider R. Schweiger S. (2007). Alternative polyadenylation signals and promoters act in concert to control tissue-specific expression of the Opitz syndrome gene MID1. BMC Mol. Biol. 8:105 10.1186/1471-2199-8-105 18005432 PMC2248598

[B218] Wood A. J. Schulz R. Woodfine K. Koltowska K. Beechey C. V. Peters J. (2008). Regulation of alternative polyadenylation by genomic imprinting. Genes Dev. 22, 1141–1146 10.1101/gad.473408 18451104 PMC2335310

[B219] Xing H. Mayhew C. N. Cullen K. E. Park-Sarge O.-K. Sarge K. D. (2004). HSF1 modulation of Hsp70 mRNA polyadenylation via interaction with symplekin. J. Biol. Chem. 279, 10551–10555 10.1074/jbc.M401221200 14707147

[B220] Yan J. Marr T. G. (2005). Computational analysis of 3′-ends of ESTs shows four classes of alternative polyadenylation in human, mouse, and rat. Genome Res. 15, 369–375 10.1101/gr.3109605 15741508 PMC551563

[B221] Yang Q. Gilmartin G. M. Doublié S. (2011). The structure of human cleavage factor I(m) hints at functions beyond UGUA-specific RNA binding: a role in alternative polyadenylation and a potential link to 5′ capping and splicing. RNA Biol. 8, 748–753 10.4161/rna.8.5.16040 21881408 PMC3256351

[B222] Yang Y. Ho S. C. L. Yap M. G. S. (2009). Mutated polyadenylation signals for controlling expression levels of multiple genes in mammalian cells. Biotechnol. Bioeng. 102, 1152–1160 10.1002/bit.22152 18973284

[B223] Yao C. Biesinger J. Wan J. Weng L. Xing Y. Xie X. (2012a). Transcriptome-wide analyses of CstF64-RNA interactions in global regulation of mRNA alternative polyadenylation. Proc. Natl. Acad. Sci. U.S.A. 109, 18773–18778 10.1073/pnas.1211101109 23112178 PMC3503179

[B224] Yao P. Potdar A. A. Arif A. Ray P. S. Mukhopadhyay R. Willard B. (2012b). Coding region polyadenylation generates a truncated tRNA synthetase that counters translation repression. Cell 149, 88–100 10.1016/j.cell.2012.02.018 22386318 PMC3615456

[B225] Yepiskoposyan H. Aeschimann F. Nilsson D. Okoniewski M. Mühlemann O. (2011). Autoregulation of the nonsense-mediated mRNA decay pathway in human cells. RNA 17, 2108–2118 10.1261/rna.030247.111 22028362 PMC3222124

[B226] Yoon O. K. Hsu T. Y. Im J. H. Brem R. B. (2012). Genetics and regulatory impact of alternative polyadenylation in human B-lymphoblastoid cells. PLoS Genet. 8:e1002882 10.1371/journal.pgen.1002882 22916029 PMC3420953

[B227] Yoshimoto K. Iwahana H. Fukuda A. Sano T. Saito S. Itakura M. (1992). Role of p53 mutations in endocrine tumorigenesis: mutation detection by polymerase chain reaction-single strand conformation polymorphism. Cancer Res. 52, 5061–5064 1516062

[B228] Yu K. Ganesan K. Tan L. K. Laban M. Wu J. Zhao X. D. (2008). A precisely regulated gene expression cassette potently modulates metastasis and survival in multiple solid cancers. PLoS Genet. 4:e1000129 10.1371/journal.pgen.1000129 18636107 PMC2444049

[B229] Zhang H. Lee J. Y. Tian B. (2005). Biased alternative polyadenylation in human tissues. Genome Biol. 6, R100 10.1186/gb-2005-6-4-r37 16356263 PMC1414089

[B230] Zhao D. Duan H. Kim Y.-C. Jefcoate C. R. (2005). Rodent StAR mRNA is substantially regulated by control of mRNA stability through sites in the 3′-untranslated region and through coupling to ongoing transcription. J. Steroid Biochem. Mol. Biol. 96, 155–173 10.1016/j.jsbmb.2005.02.011 16039847

[B231] Zhao Y. Koga K. Osuga Y. Nagai M. Izumi G. Takamura M. (2012). Thrombin enhances soluble Fms-like tyrosine kinase 1 expression in trophoblasts; possible involvement in the pathogenesis of preeclampsia. Fertil. Steril. 98, 917–921 10.1016/j.fertnstert.2012.04.014 22819145

[B232] Zhou H.-L. Baraniak A. P. Lou H. (2007). Role for Fox-1/Fox-2 in mediating the neuronal pathway of calcitonin/calcitonin gene-related peptide alternative RNA processing. Mol. Cell. Biol. 27, 830–841 10.1128/MCB.01015-06 17101796 PMC1800674

[B233] Zhou L. Yuan Q. Yang M. (2012). A functional germline variant in the P53 polyadenylation signal and risk of esophageal squamous cell carcinoma. Gene 506, 295–297 10.1016/j.gene.2012.06.089 22800615

[B234] Zhu H. Zhou H.-L. Hasman R. A. Lou H. (2007). Hu proteins regulate polyadenylation by blocking sites containing U-rich sequences. J. Biol. Chem. 282, 2203–2210 10.1074/jbc.M705064200 17127772

[B235] Zhu Z.-H. Yu Y. P. Shi Y.-K. Nelson J. B. Luo J.-H. (2009). CSR1 induces cell death through inactivation of CPSF3. Oncogene 28, 41–51 10.1038/onc.2008.359 18806823 PMC2918272

